# Role of Membrane Estrogen Receptor Alpha on the Positive Feedback of Estrogens on Kisspeptin and GnRH Neurons

**DOI:** 10.1523/ENEURO.0271-23.2024

**Published:** 2024-10-18

**Authors:** Mélanie C. Faure, Rebeca Corona, Céline Roomans, Françoise Lenfant, Jean-Michel Foidart, Charlotte A. Cornil

**Affiliations:** ^1^Laboratory of Neuroendocrinology, GIGA Neurosciences, University of Liège, Liège, Belgium; ^2^Institute of Metabolic and Cardiovascular Diseases (I2MC) Equipe 4, Inserm U1297-UPS, CHU, Toulouse 31432, France; ^3^Department of Obstetrics and Gynecology, University of Liège, Liège, Belgium; ^4^Estetra SRL, Légiapark, Boulevard Patience et Beaujonc 3, 4000 Liège, Belgium

**Keywords:** estetrol, GnRH neurons; kisspeptin neurons, LH surge, mERα, preoptic area

## Abstract

Estrogens act through nuclear and membrane-initiated signaling. Estrogen receptor alpha (ERα) is critical for reproduction, but the relative contribution of its nuclear and membrane signaling to the central regulation of reproduction is unclear. To address this question, two complementary approaches were used: estetrol (E_4_) a natural estrogen acting as an agonist of nuclear ERs, but as an antagonist of their membrane fraction, and the C451A-ERα mouse lacking mERα. E_4_ dose- dependently blocks ovulation in female rats, but the central mechanism underlying this effect is unknown. To determine whether E_4_ acts centrally to control ovulation, its effect was tested on the positive feedback of estradiol (E_2_) on neural circuits underlying luteinizing hormone (LH) secretion. In ovariectomized females chronically exposed to a low dose of E_2_, estradiol benzoate (EB) alone or combined with progesterone (P) induced an increase in the number of kisspeptin (Kp) and gonadotropin-releasing hormone (GnRH) neurons coexpressing Fos, a marker of neuronal activation. E_4_ blocked these effects of EB, but not when combined to P. These results indicate that E_4_ blocked the central induction of the positive feedback in the absence of P, suggesting an antagonistic effect of E_4_ on mERα in the brain as shown in peripheral tissues. In parallel, as opposed to wild-type females, C451A-ERα females did not show the activation of Kp and GnRH neurons in response to EB unless they are treated with P. Together these effects support a role for membrane-initiated estrogen signaling in the activation of the circuit mediating the LH surge.

## Significance Statement

Estrogen receptor alpha (ERα) is critical for the activation of the neural circuits underlying ovulation. However, the relative contribution of its nuclear and membrane signaling to this neuroendocrine phenomenon is unclear. Using two complementary approaches to block membrane ERα signaling, the present study reveals that membrane ERα signaling is required for the activation by estrogens of gonadotropin-releasing hormone (GnRH) and kisspeptin (Kp) neurons, two key neuronal populations underlying the surge of luteinizing hormone which triggers ovulation. Interestingly, the absence of activation of Kp and GnRH neurons is alleviated in both models by progesterone (P). Collectively the results of these two approaches converge to provide evidence that membrane estrogen signaling contributes to this key event for the central regulation of reproduction.

## Introduction

Gonadotropin-releasing hormone (GnRH) neurons stand at the top of the hypothalamus–pituitary–gonadal (HPG) axis that governs reproduction. Their activity drives the pulsatile release of gonadotropins to govern ovarian steroidogenesis and folliculogenesis. During most of the cycle, estrogens exert a negative feedback on GnRH and gonadotropin secretion. At mid-cycle, estrogens switch from negative to positive feedback to generate a continuous surge of GnRH and subsequently a luteinizing hormone (LH) surge which triggers ovulation (Herbison, 1998, 2020; Wang and Moenter, 2020). The mechanisms underlying the action of estrogens leading to the initiation of the preovulatory LH surge remain however unclear.

The nuclear estrogen receptor alpha (nERα) is the primary estrogen receptor (ER) involved in the central control of reproduction (Hamilton et al., 2014). As GnRH neurons do not express ERα (Herbison and Pape, 2001), the positive feedback is mediated by ERα-expressing afferents to GnRH neurons mainly originating from the anteroventral periventricular nucleus (AVPv; Wintermantel et al., 2006; Campbell and Herbison, 2007). In particular, kisspeptin (Kp) neurons exert a pivotal role in translating changes in circulating estrogens into changes in the activity of GnRH neurons and LH surge generation (Wang et al., 2016, 2018; Porteous and Herbison, 2019). Although other neuronal populations likely contribute to the estrogenic regulation of GnRH neurons, the current view posits Kp neurons located in the AVPv as key elements of the core surge generator (Goodman et al., 2022).

Estrogens act through nuclear and membrane-initiated signaling. Nuclear signaling regulates the transcription of target genes through direct interaction of the liganded receptor with an estrogen response element (ERE; classical genomic action) on the DNA or via protein–protein interaction with another transcription factor (tethered genomic action; McDevitt et al., 2008). Upon palmitoylation, ERs are translocated to the membrane where they can signal to activate intracellular signaling cascades (Arnal et al., 2017; Acconcia et al., 2021). Additionally, estrogens also act on membrane-specific G-protein-coupled receptors such as GPER1 (Kelly and Rønnekleiv, 2015). While nuclear actions lead to relatively slow and long-lasting effects, membrane-initiated actions occur within seconds to minutes (Kelly and Rønnekleiv, 2015; Balthazart, 2021).

Whether the central regulation of LH surge involves nuclear- or membrane estrogen-initiated signaling or a combination of both is currently unclear. Early evidence indicated that a prolonged exposure to high circulating estrogens is required to elicit an LH surge (Legan et al., 1975; Evans et al., 1997), suggesting that classical estrogen signaling is involved. This is supported by reports indicating that ERE-independent ERα activity alone is not sufficient to restore E_2_-induced changes in the firing rate of GnRH neurons or the LH surge (Glidewell-Kenney et al., 2007; Christian et al., 2008). However, that transcriptional signaling is required does not preclude a role of membrane-initiated signaling. Moreover, membrane-initiated estrogen signaling also influences GnRH neurons in vitro (Herbison, 2009; Moenter and Chu, 2012; Terasawa and Kenealy, 2012). While ERβ (Abraham et al., 2003; Chu et al., 2009) or membrane-specific estrogen receptors, such as the STX-activated receptor (Zhang et al., 2010) or GPER1 (Sun et al., 2010), appear to mediate a direct action of estrogens on the activity of GnRH neurons, ERα would mediate indirect estrogenic actions by modulating inputs to GnRH neurons (Romano et al., 2008; Chu et al., 2009; Romanò and Herbison, 2012). In particular, membrane ERα (mERα) stimulates neuronal activity and contributes to the regulation of Kp expression in immortalized Kp neurons with features of AVPv Kp neurons (Mittelman-Smith et al., 2015). Evidence obtained in vitro also indicates that the activation of mERα mediates the synthesis of neuroprogesterone by rostral hypothalamic astrocytes (Micevych et al., 2007; Kuo et al., 2010; Mohr et al., 2022), whose action on Kp neurons is necessary for both Kp release (Mittelman-Smith et al., 2018; Mohr et al., 2021) and LH surge induction (Micevych et al., 2003; Mohr et al., 2019; Chuon et al., 2022). Thus, mERα signaling appears to be able to modulate the activity of AVPv Kp neurons both directly and indirectly in vitro. Yet, to our knowledge, whether direct or indirect, a role for membrane estrogen signaling on the activation of Kp neurons and the subsequent activation of GnRH neurons has never been demonstrated in vivo.

The present study took advantage of genetic and pharmacological complementary approaches to explore the role of mERα on the central regulation of LH surge. First, a knock-in mouse model with a point mutation of the palmitoylation site Cys451 into an alanine leads to a selective loss of function of ERα membrane signaling, allowing to dissociate the two modes of action of estrogens on ERα (Adlanmerini et al., 2014; Pedram et al., 2014). Second, estetrol (E_4_) is an estrogen exclusively synthesized in human fetal liver which selectively binds ERα and ERβ with a lower affinity than E_2_ (Holinka et al., 2008). E_4_ presents unique properties allowing to distinguish nuclear and membrane estrogen signaling in rodents notably, as it mimics estrogenic actions induced via the activation of nuclear ERα but antagonizes membrane ERα in different tissues (Gérard et al., 2022). E_4_ inhibits ovulation when administered alone in rats (Coelingh Bennink et al., 2008) and when combined with progesterone (P) in humans (Duijkers et al., 2015; Apter et al., 2016). E_4_ is now included in an oral contraceptive formulation (Klipping et al., 2021). However, its central mechanism of action on the HPG axis remains unknown.

## Materials and Methods

### Animals and general procedures

All wild-type (WT-ERα) and C451A-ERα mice of the CD1 strain, obtained by backcrossing the original C451A-ERα mice (C57Bl/6) into the CD1 background (Adlanmerini et al., 2014), were housed and bred in the animal facility of the University of Liège. Mice were genotyped by PCR analysis of DNA collected from the tail as described previously (Adlanmerini et al., 2014). Mice were weaned at 3–4 weeks of age and housed in same-sex cages. All animals had *ad libitum* access to food and water. The room temperature was maintained at 24 ± 2°C. Animals were housed under a reversed 12 h light/dark cycle (lights on at 1 A.M.) when tested for positive feedback (Exp. 1 and 2). All experimental procedures were in accordance with laws on the “Protection and Welfare of Animals” and on the “Protection of Experimental Animals” and were approved by the Ethics Committee of the University of Liège.

### General procedures

#### Surgery

Between 2 and 3 months of age, females were bilaterally ovariectomized (OVX) under general anesthesia using a mixture of Domitor (Domitor, Pfizer, 1 mg/kg) and medetomidine (Ketamine, 80 mg/kg) administered subcutaneously (s.c.). In some experiments, animals were implanted at the time of ovariectomy with a subcutaneous Silastic capsule filled with E_2_. At the end of surgery, medetomidine-induced effects were antagonized by atipamezole (Antisedan, Pfizer, 4 mg/kg, s.c.) to accelerate recovery.

#### Hormones

17β-Estradiol (E_2_, E8875), β-estradiol-3-benzoate (EB, E8515), and progesterone (P, P0130) were purchased from Sigma-Aldrich and dissolved in sesame oil, used as vehicle, unless stated otherwise. EB (1 µg, s.c.) and P (500 µg, s.c.) were injected subcutaneously, while E_2_ (1 µg diluted in 7.35 µl of sesame oil/20 g of body weight) was provided through subcutaneous Silastic capsules (inner diameter, 1.02 mm; outer diameter, 2.16 mm; Dow Corning) which yield physiological circulating E_2_ concentration (Bronson, 1981). Estetrol (E_4_) was provided by Mithra Pharmaceuticals and dissolved in sesame oil with 5% ethanol (0.2 mg, 50 µl, s.c.). Unless stated otherwise, treatments were counterbalanced across housing cages, such that each/every cage contained animals with different treatments.

#### Blood collection

Depending on the question and the method used for blood analysis, blood drops or trunk blood were collected. For repeated sampling of blood drops on a same day or assay with ultrasensitive immune-enzyme assays [EIA; Exp. 1-part1 (1.1)], blood was collected using the repetitive tail-tip blood sampling (Czieselsky et al., 2016). Briefly, mice were habituated to handling for a few minutes while massaging the tail every day during 2 or 3 weeks. For blood drop collection, a single excision of the tail tip was made with a razor blade. When females were OVX (regardless of whether they were treated with EB and/or P), one blood sample (5.2 µl) was collected with a pipette and immediately diluted in 98.8 µl phosphate-buffered saline with 0.05% of Tween 20 (PBST), quickly frozen in dry ice, and stored at −80°C until further use. In Exp. 1.1, blood drops were collected every 30 min for 4 h. For Exp. 1.2, mice were placed under a red lamp to allow dilation of blood vessels and were briefly restrained in the immobilizing cage where a single excision of the tail with a razor blade was made. Blood (200 µl) was collected in heparinized microhematocrit capillary tubes filled by capillarity. The tail was massaged to facilitate blood dripping. Blood was stored in a 1.5 ml microfuge tube containing a drop of heparin (Leo, 012866-08, 5,000 U.E/ml). Blood was centrifuged 10 min at 1,500 × *g* at 4°C, the plasma was collected and stored at −80°C until quantification by radioimmunoassay (RIA). At the end of experiments (Exp. 1.2 and Exp. 2), trunk blood was also collected in 1.5 ml microfuge tubes containing a drop of heparin. Plasma was collected as previously and stored at −80°C until further use.

#### LH assay

Two methods were used to assay LH: an ultrasensitive sandwich ELISA and a classical RIA. The ultrasensitive sandwich ELISA was used for blood drops [Exp. 1.1 and 1.2 (day 39)], while the RIA was used for all the other types of blood samples (Exp. 1.2 and Exp. 2).

We used the sensitive sandwich ELISA previously described and validated (Steyn et al., 2013) with few modifications. Briefly, 96-well high-affinity binding microplates (9018, Corning) were coated with 50 µl of a monoclonal antibody directed against bovine LH beta subunit (1:1,000; 518B7; RRID: AB_2665514, University of California, UC Davis) and incubated overnight at 4°C. Unspecific binding was blocked by incubating each well with 200 µl of blocking buffer for 24 h at 4°C. Samples (50 µl) and LH standards [50 µl; generated by serial twofold dilution of mouse LH starting at 400 pg/well until 0,19 pg/well, AFP-5306A, National Institute of Diabetes and Digestive and Kidney Diseases – National Hormone and Pituitary Program (NIDDK-NHPP)] were incubated for 2 h before adding 50 µl of detection antibody (1:10,000; polyclonal antibody, rabbit LH antiserum, AFP240580Rb; RRID:AB_2665533, NIDDK-NHPP) for 1.5 h at room temperature (RT). A horseradish peroxidase-conjugated polyclonal Goat Anti-Rabbit antibody (50 µl, 1:2,000; P0448, Dako; RRID:AB_2617138) was added in each well for 1.5 h at RT. Then, the substrate of the peroxidase (100 µl, 3,3′,5,5′-tetramentylbenzidine solution; 1-Step Ultra TMB-ELISA, 34029, Thermo Fisher Scientific) was added in each well for 10 to 25 min at RT and in darkness. The reaction was stopped by 3 M HCl (50 µl). The absorbance of each well was read at a wavelength of 450 nm and at a wavelength of 650 nm (background). The optical density (OD) obtained at 650 nm was subtracted from this obtained at 450 nm. The amount of LH present in each well was determined by interpolating the resulting OD of unknown samples against a nonlinear regression of the OD of the LH standard curve (GraphPad Prism 8). Standards were run in duplicate and yielded a nonlinear curve fitting with a *R*^2 ^> 0.95. The sensitivity of the assay was 0.03 ng/ml. All samples from a same mouse were assayed on the same plate, and genotypes and treatments were counterbalanced within plates. The intra- and inter-assay coefficients of variation were <10 and 15%, respectively.

The RIA consisted of a double antibody method with reagents provided by the National Institutes of Health [Dr. A. F. Parlow, National Institute of Diabetes and Digestive and Kidney Diseases (NIDDK), National Hormone and Peptide Program, Torrance, CA]. LH was detected by a rat LH-I-10 (AFP-11536B) labeled with ^125^I and precipitated with a Rabbit anti-mouse LH (AFP-240580; RRID: AB_2784499). Mouse LH reference preparation (AFP-5306A) was used to prepare the standard curve. The intra- and inter-assay coefficients were <10 and 7%, respectively, and the sensitivity of the method was set at 4 pg/100 ml based on the lowest detectable point of the standard curve.

The values of LH concentrations obtained for each animal on the day of LH induction were compared with the average values measured in all samples within each genotype collected on the morning of the day preceding the LH surge induction. This average plus two times the standard deviation was considered as the threshold for considering an LH surge (Dror et al., 2013). The percentage of animals that presented a surge was then calculated for each group.

#### Euthanasia

Animals were humanely anesthetized with isoflurane and decapitated 30 min after lights off (Exp. 1 and 2). Their brain was then removed from the skull and immersed in a solution of 0.5% of acrolein in 0.01 M PBS for 2 h at RT. For this type of fixation, brains were rinsed thrice for 30 min in PBS before being transferred in 30% sucrose overnight. Brains were then frozen on dry ice and stored at −80°C until further use. All brains were cryosectioned in four series of 30 µm thick coronal slices from the corpus callosum level to the end of the hypothalamus. Sections were stored in antifreeze solution and kept at −20°C.

#### Histology and immunostaining

Brains were double labeled for Fos and Kp or GnRH. Briefly, brain sections were first rinsed three times for 5 min in 0.05 M Tris-buffered saline (TBS), pH 7.6, at RT. Unless mentioned otherwise, all following incubations were carried out at RT and followed by similar rinses. Sections were first incubated in 0.1% sodium borohydrate for 15 min. They were then incubated in hydrogen peroxide (H_2_O_2_, 1% for 20 min) to block endogenous peroxidase activity. Sections were blocked and permeabilized for 1 h in normal goat serum (NGS) in TBS with 0.1% Triton X-100 (TBST) and immediately incubated at 4°C in the primary antibody against the N terminus of human Fos [overnight, 1:2,000; Rabbit polyclonal, ABE457, Millipore; RRID: AB_2631318 (Alvisi et al., 2016; Exp. 1.2); overnight, 1:2,000; monoclonal antibody, sc-166940, Santa Cruz Biotechnology; RRID: AB_10609634 (Exp. 2)] in NGS and TBST. Sections were then incubated for 2 h in a goat anti-rabbit biotinylated antibody (111-065-003; RRID: AB_2337959; Jackson ImmunoResearch) followed by 1 h in the AB complex solution (PK-6100; Vector Laboratories) diluted at 1:400 or 1:800 (for Fos when followed by GnRH labeling or for Fos when followed by Kp labeling, respectively). The immunoproduct was visualized with 0.05% diaminobenzidine with 0.012% H_2_O_2_ in TBS.

The first visualization was followed by a blockade of avidins and biotins using avidin-biotin blocking kit (SP-2001; Vector Laboratories) for 15 min prior to an additional blocking and permeabilization step. Sections were immediately incubated overnight in a polyclonal rabbit antibody directed against GnRH-I [1:400, polyclonal, #20075, Immunostar; RRID: AB_572248 (Memi et al., 2013)] or twice overnight in a rabbit antibody directed against mouse Kp [1:10,000; rabbit polyclonal, Ac566 kindly provided by Isabelle Franceschini and Massimiliano Beltramo, INRA, Nouzilly, Tours, France; RRID: AB_2296529 (Clarkson and Herbison, 2006)] in NGS and TBST. Sections were then incubated in a goat anti-rabbit biotinylated secondary antibody (111-065-003; Jackson ImmunoResearch). Finally, the immunoproduct was visualized by a last incubation in the substrate of the Vector SG Peroxidase Substrate Kit (SK-4700; Vector Laboratories). After final rinses, sections were mounted on microscope slides and coverslipped with Eukitt (Sigma-Aldrich).

#### Image analysis

The number of single-labeled Kp-immunoreactive (IR) neurons or the number of Kp-IR and GnRH-IR neurons colabeled with Fos was analyzed by direct observation at 40× magnification using Leica DMRB microscope. The number of Kp-IR cell bodies was investigated bilaterally in 10 consecutive brain sections (each separated by a distance of 90 µm) encompassing the AVPv and the rostral periventricular nucleus (PeN) continuum [corresponding to plates 29–35 of the Paxinos Mouse Atlas (Franklin and Paxinos, 2001)]. The number of GnRH-IR cell bodies was analyzed bilaterally in 10 consecutive brain sections (each separated by a distance of 90 µm) corresponding to plates 21–31 of the Paxinos Mouse Atlas (Franklin and Paxinos, 2001). Kp and GnRH immunolabeling is cytoplasmic, while Fos immunolabeling is detectable only in the nucleus. All Kp or GnRH neurons detected in this region were counted and analyzed for the presence of nuclear immunostaining for Fos. The values obtained for each side of the 10 sections were summed to provide a total number of Kp or GnRH expressing neurons and the percentage of Kp or GnRH neurons coexpressing the protein Fos.

### Experimental designs

#### Experiment 1—positive feedback

**Figure 1.**
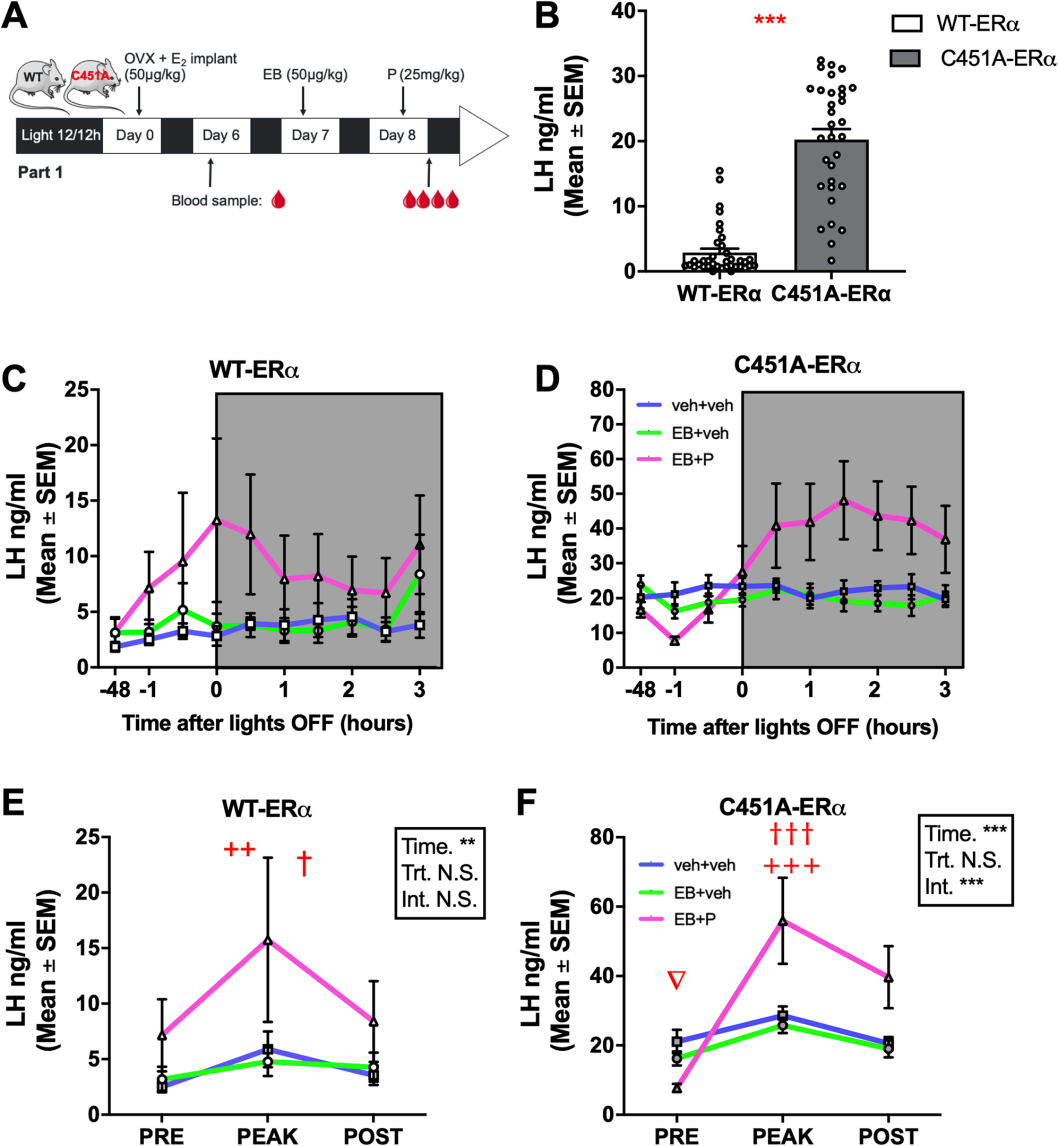
Profiles of LH changes induced by estradiol benzoate (EB) alone or in combination with progesterone (P) in ovariectomized WT-ERα (white) or C451A-ERα (gray mice). ***A***, Protocol used to induce a positive feedback: females were ovariectomized (OVX), chronically treated with estradiol (E_2_) from day 0 to day 8, injected with estradiol benzoate on day 7, and injected with progesterone or its vehicle (sesame oil) on day 8. ***B***, On day 6, C451A-ERα females (*n* = 32) showed higher baseline LH levels than WT-ERα females (*n* = 31; Mann–Whitney test). ***C***, ***D***, Profiles of LH levels measured every 30 min starting 1 h before lights off following treatment on day 8 in WT-ERα and C451A-ERα females, respectively. ***C***, LH profiles obtained in WT-ERα mice (OVX + E_2 _+ veh + veh *n* = 12, OVX + E_2 _+ EB + veh *n* = 11, OVX + E_2 _+ EB + P *n* = 14). ***D***, LH profiles obtained in C451A-ERα mice (OVX + E_2 _+ veh + Veh *n* = 10, OVX + E_2 _+ EB + veh *n* = 11 OVX + E_2 _+ EB + P *n* = 12). ***E***, Regardless of treatment, WT-ERα females showed an increased LH concentration at one time point (peak) between 0 and 2.5 h after lights off compared with prior (day 6, pre) and during 3 h after lights off (post; two-way ANOVA; Tukey’s post hoc test following significant time effect: ^++^*p* < 0.01 vs “pre”; ^†^*p* < 0.05 vs “post”). ***F***, EB + P induced an increased LH concentration in C451A-ERα females within 0 and 2.5 h after lights off (peak) compared with prior (day 6, pre) and during 3 h after lights off (post; two-way ANOVA; Tukey’s post hoc test, following significant interaction: ^+++^*p* = 0.001 vs “pre” within same treatment; ^†††^*p* = 0.001 vs “post“ within same treatment; ^∇^*p* < 0.05 EB + P “pre” vs “post” within same treatment. Symbols in the statistical boxes: *, **, ***, *p* < 0.05, 0.01, 0.001; N.S., nonsignificant).

The role of mERα in the induction of LH surge was repeatedly assessed in two cohorts of 2-month-old WT-ERα (Cohort 1: *n* = 24; Cohort 2: *n* = 17) and C451A-ERα (Cohort 1: *n* = 18; Cohort 2: *n* = 19) females. The two cohorts were subjected to the exact same protocol except that females from the second cohort were housed based on their treatment. In each cohort, females were tested twice following a paradigm of LH surge induction, i.e., by implantation of a subcutaneous capsule delivering low levels of E_2_ mimicking diestrus levels and administration of EB 7–8 d after OVX (Fig. 1*A*). The first test was designed to examine the time-response profile of the EB-induced LH surge following blood sampling every 30 min for 4 h [Part 1 (Exp. 1.1), Days 0–8], while the second investigated the central activation of the circuit underlying the LH surge [Part 2 (Exp. 1.2), Days 30–39].

Briefly, females were OVX and implanted with a subcutaneous capsule containing E_2_ (1 µg). A first blood sample was collected on Day 6 post-OVX between 08.20 A.M. and 09.00 A.M. (3 µl immediately diluted in 57 µl of PBST for EIA). Females of each genotype were subdivided in three groups of equal size subjected to three different hormonal treatments (s.c.): veh + veh, EB + veh, and EB + P. On Day 7 (10 A.M.), they were injected with EB or its vehicle (veh). On Day 8 (10 A.M.), they were injected with P or veh 3 h before lights off, while females that had received veh on Day 6 received veh again. Blood sampling was then carried out every 30 min for 4 h starting 60 min before lights off. All samples were assayed in duplicate. Three to 7 d later, their implant was removed, and they were treated every 3 or 4 d with EB until the beginning of the second part.

**Figure 2.**
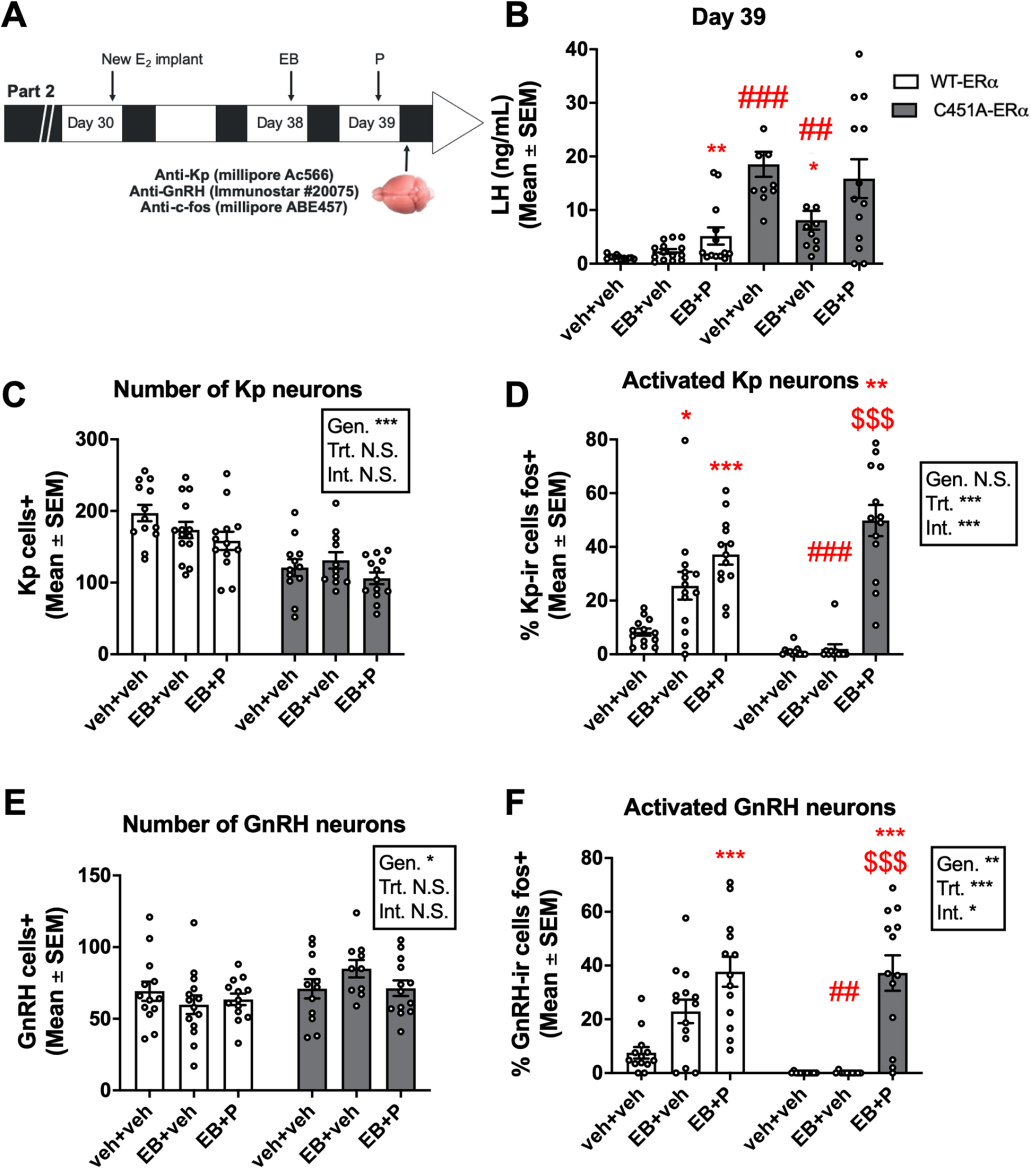
Effect of mERα absence on the positive feedback of estrogens on LH concentration and the activation of the associated neurocircuits. ***A***, Protocol used to induce positive feedback: following a first round of injections to induce the positive feedback (Fig. 1), the E_2_ implant was replaced by a new one on Day 30, and females were treated again with veh + veh, EB + veh, or EB + P on Days 38 and 39. Blood and brains were collected 30–60 min after lights off. ***B***, In WT-ERα females (white), EB + P, but not EB + veh, induced a significant rise in LH (Kruskal–Wallis test: **p < 0.01 vs veh + veh), while in C451A-ERα females (gray), EB + veh induced a significant reduction in LH (Kruskal–Wallis test: *p < 0.05 vs veh + veh; Mann–Whitney tests: ##, ### < 0.01, 0.001 vs WT-ERα within same treatment). ***C***, WT-ERα females displayed more kisspeptin (Kp) neurons in RP3 V (AVPv + PeN) than C451A-ERα females (two-way ANOVA). ***D***, A higher percentage of Kp neurons coexpressed Fos following EB and EB + P than veh + veh in WT-ERα, while only EB + P induced such activation in C451A-ERα (two-way ANOVA; * and ***, *p* < 0.05 and 0.001 vs veh + veh same genotype; ^$$$^*p* < 0.0001 vs EB + veh same genotype; ^###^*p* < 0.001 vs same treatment in WT-ERα). ***E***, GnRH neurons counted in POA were slightly more abundant in C451A-ERα females than in WT-ERα females (two-way ANOVA). ***F***, A higher percentage of GnRH neurons coexpressed Fos following EB and EB + P than veh + veh in WT-ERα, while only EB + P induced such activation in C451A-ERα (two-way ANOVA; ***, *p* < 0.001 vs veh + veh same genotype; ^$$$^*p* < 0.0001 vs EB + veh same genotype; ^##^*p* < 0.01 vs same treatment in WT-ERα). Sample size: ***B***, ***C***. WT-ERα: veh + veh, *n* = 14, veh + EB, *n* = 14, EB + P, *n* = 13, C451A-ERα: veh + veh, *n* = 11, veh + EB, *n* = 11, EB + P, *n* = 13. ***C***–***F***. WT-ERα: veh + veh, *n* = 13, veh + EB, *n* = 14, EB + P, *n* = 13, C451A-ERα: veh + veh, *n* = 12, veh + EB, *n* = 11, EB + P, *n* = 13. Symbols in the statistical boxes: *, **, ***, *p* < 0.05, 0.01, 0.001; N.S., nonsignificant.

Part 2 started 30 d after Part 1. Females were reimplanted with a new subcutaneous E_2_ implant. Two blood samples were collected on Day 38 between 8 A.M. and 9 A.M.: 5.2 µl immediately diluted in PBST for EIA and 200 µl for plasma collection and RIA. Females were then treated with veh or EB at ∼10 A.M. The next day (Day 39), veh or P was injected 3 h before lights off. Mice were anaesthetized with isoflurane 30 min after lights went off and killed by rapid decapitation. Trunk blood was collected, extracted for plasma as described above, and assayed by EIA. Brains were fixed in 0.5% acrolein (Fig. 2*A*).

#### Experiment 2—E_4_ and positive feedback

**Figure 3.**
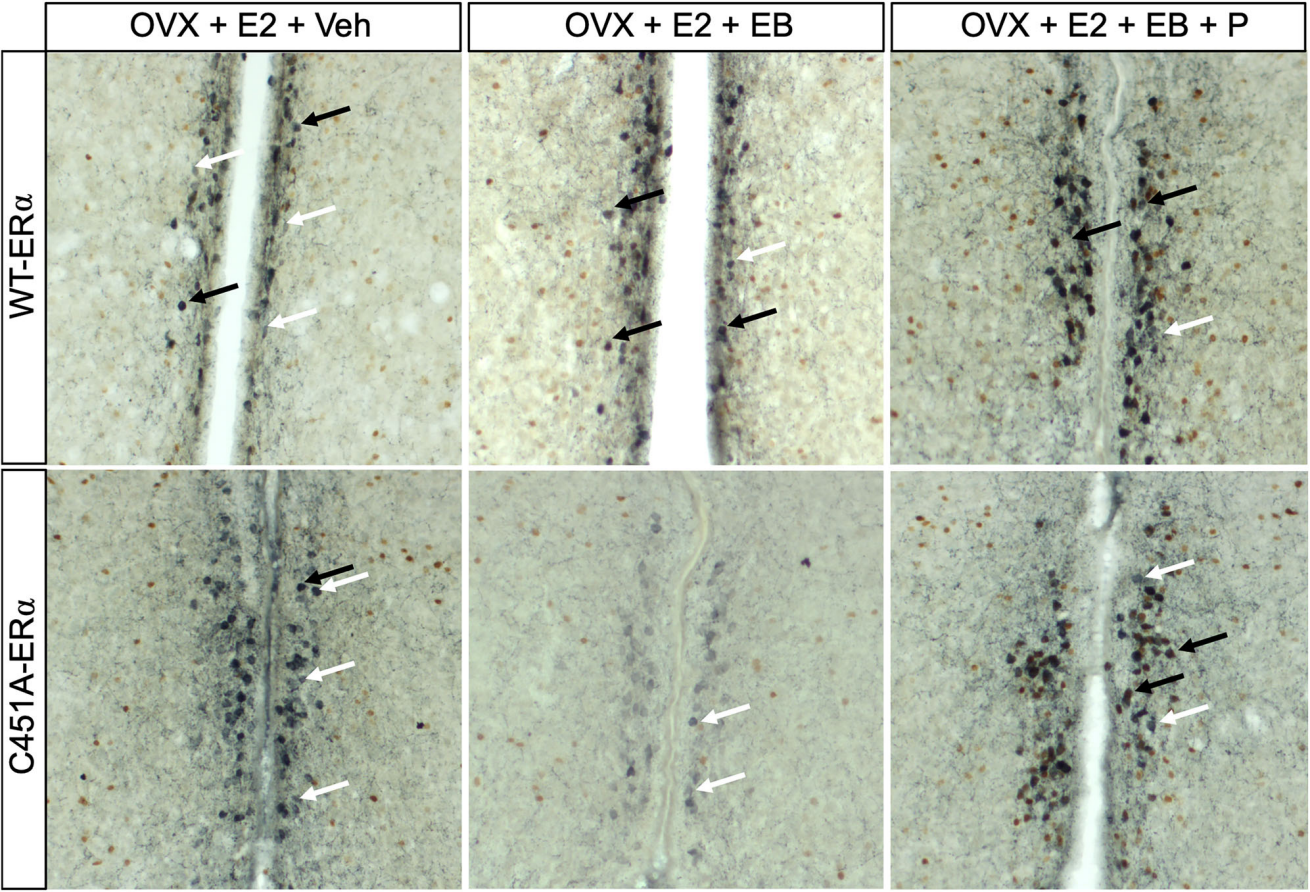
Representative photomicrographs of Kp-IR neurons (in blue) and their coexpression of the neuronal activity marker Fos (in orange) as a function of the treatment and genotype. Black arrows point at double-labeled neurons, while white arrows point at single-labeled neurons.

This experiment investigated the effect of E_4_ on the induction of LH surge in WT-ERα females (*n* = 45) subjected to a classical paradigm of induction of the LH surge by administration of EB with or without P in OVX females chronically exposed to low estrogen levels mimicking diestrus levels (Fig. 3*A*). Briefly, females were OVX and implanted with a subcutaneous E_2_ capsule. Prior to treatment, one blood sample (200 µl) was collected on Day 8 after OVX. Females were subdivided into five groups and subjected to five different hormonal treatments: veh + P, EB + veh, EB + P, EB + E_4_, and EB + E_4 _+ P. On Day 8 (10 A.M.), they were injected with veh, EB, or EB + E_4_. On Day 9 (10 A.M.), they were injected with P or veh 4 h before lights off. Females were killed by rapid decapitation within 1 h after lights off, trunk blood was collected, and the brain was dissected out of the skull and fixed in 0.5% acrolein.

#### Statistical analysis

All statistical analyses were performed using Prism 8 (version 8.0.0, GraphPad Software). Continuous data were analyzed by parametric unpaired Student’s *t* tests and two-way ANOVAs or by nonparametric Mann–Whitney and Kruskal–Wallis tests when the normality and homoscedasticity assumptions were violated. Significant parametric and nonparametric ANOVAs were followed by Tukey' and Dunn’s post hoc tests, respectively. Contingency data were analyzed by Fisher’s exact tests. Bonferroni’s correction was applied when multiple Mann–Whitney tests were applied to a data set. The resulting *p* value is then called adjusted *p* value (*p*_adj_). Due to technical issues such as the loss or the degradation of sections during processing, the final sample size may differ from the initial number of samples collected, thus explaining the variability in the degrees of freedom between analyses of samples originating from the same experiments. Effects sizes from ANOVA (partial eta squares, η_p_^2^) were calculated based on the sums of squares provided by the ANOVAs or using calculators available at https://www.psychometrica.de/effect_size.html for Kruskal–Wallis analyses. Effect sizes for Student’s *t* or Mann–Whitney test (Cohen’s *d*) were obtained using calculators available at https://wwhttps://www.psychometrica.de/effect_size.html. Results were considered significant when *p* < 0.05. All results are represented as means ± SEM unless mentioned otherwise.

## Results

### Are C451A-ERα mice able to show an LH surge in response to EB and is P necessary?

Although the paradigm of rising E_2_ levels can induce an LH surge in the absence of P, the combination of E_2_ and P yields changes of higher amplitude (Bronson and Vom Saal, 1979; Waring and Turgeon, 1992). Therefore, the first experiment investigated the role of mERα on the LH surge profile induced by EB combined or not with P. OVX females were implanted with a capsule delivering low E_2_ amounts mimicking circulating E_2_ levels at diestrus (Dror et al., 2013), and blood was collected by tail-tip blood sampling every 30 min for 4 h starting 1 h prior to lights off (Exp. 1). This experiment was conducted in two cohorts of mice, subjected to the exact same protocol, whose data were pooled. First, looking at baseline LH levels (6 d after OVX and implantation of a subcutaneous capsule delivering low levels of E_2_), C451A-ERα females showed significantly higher LH levels than their WT-ERα littermates (WT-ERα, median = 1.4 ng/ml, *n* = 42; C451A-ERα, median = 21.6 ng/ml, *n* = 36; *U* = 47, *p* < 0.0001, *d* = 2.474; Fig. 1*B*), indicating that C451A-ERα females may present some impairment of the negative feedback.

The qualitative analysis of the average profiles of LH concentration measured every 30 min on Day 8 indicates that treatment with EB + P resulted in an increased LH concentration, while no surge was induced neither in the control condition (veh + veh) nor following EB alone in both WT-ERα and C451A-ERα mice (Fig. 1*C*,*D*). Of note, in WT-ERα, LH began to rise before lights off, peaked at lights off, and slightly decreased afterward while remaining elevated for the next 3 h (Fig. 1*C*), while in C451A-ERα mice, the LH surge began with a slight delay compared with WT-ERα, peaked 30 min after lights off and remained elevated for the next 2.5 h (Fig. 1*D*). Interestingly, in C451A-ERα females treated with EB + P and EB + veh, the first time point (−1 h) shows a clear decrease in LH concentration compared with the measure taken 48 h earlier (day 6) potentially reflecting a negative feedback exerted by EB. In both genotypes, there is a large variability around the mean for most time points which is explained by the variability in individual profiles. Additional work is warranted to confirm the existence of a delay in the response of C451A-ERα females.

For analysis purposes, the highest LH concentrations obtained in each animal between 0 and 2.5 h after lights off (Peak) were averaged across females and compared with the concentration measured 48 h before (Pre) and 3 h after (Post) lights off (Fig. 1*E*,*F*). Confirming the qualitative observations, no LH surge was observed following treatment with veh or EB alone in both genotypes. In WT-ERα, the analysis revealed no effect of treatment (*F*_(2,33) _= 1.488; *p* = 0.2405; $\eta_{p}^{2}$ = 0.345), but a time effect (*F*_(2,66) _= 6.747; *p* = 0.0022; $\eta_{p}^{2}$ = 0.034; Fig. 1*E*) which results from a higher LH level measured at the peak compared with the pre (*p* = 0.022) and post conditions (*p* = 0.026). Despite the marked increase in LH exhibited by females treated with EB + P, there was no interaction (*F*_(4,66) _= 1.797; *p* = 0.1400; $\eta_{p}^{2}$ = 0.098). In C451A-ERα, the analysis revealed no effect of treatment (*F*_(2,30) _= 2.087; *p* = 0.1417; $\eta_{p}^{2}$ = 0.243), but a time effect (*F*_(2,60) _= 19.04; *p* < 0.0001; $\eta_{p}^{2}$ = 0.216; Fig. 1*F*) and an interaction between the two factors (*F*_(4,60) _= 8.519; *p* < 0.0001; $\eta_{p}^{2}$ = 0.362). These effects are explained by significant differences between all time points in EB + P treated females only (Tukey’s post hoc test, *p* < 0.0135, “peak” vs other time points). Therefore, despite elevated LH basal levels, C451A-ERα mice appear able to mount a LH surge.

Three weeks later, the same mice were then subjected to the same protocol with minor changes. Their blood and brain were collected between 30 min and 1 h after lights off to evaluate the impact of the mutation on the neuronal circuits underlying the induction of a LH surge by estrogens. As before, C451A-ERα showed higher LH concentrations than WT-ERα prior to EB (WT-ERα: 0.95 ng/ml ±0.17, *n* = 16, C451A-ERα: 14.89 ng/ml ±1.80, *n* = 15; *t*_(29) _= 7.952, *p* < 0.001, *d* = 2.858). The analyses of blood samples collected at euthanasia identified an increase in LH in WT-ERα females treated with EB + P, but not with veh + EB compared with veh + veh (*H* = 10.02; *p* = 0.0067; $\eta_{p}^{2}$ = 0.211; Fig. 2*B*). In contrast, although LH significantly decreased after EB alone, there was no effect of EB + P in C451A-ERα females (*H* = 7.301, *p* = 0.0260, $\eta_{p}^{2}$ = 0.156; veh + veh vs EB + veh, *p* = 0.0145; Fig. 2*B*). Comparisons between genotypes in each condition confirmed the higher LH levels measured in C451A-ERα compared with WT-ERα females in all conditions, but not in EB + P condition (veh + veh: *U* = 0, *p*_adj _< 0.0003, *d* = 3.191; EB + veh: *U* = 21, *p*_adj _= 0.0042, *d* = 1.552; EB + P, *U* = 44, *p*_adj _= 0.1137, *d* = 0.892). Accordingly, the analyses of the percentages of females presenting a surge indicate that WT females treated with EB + P (62%; *p* = 0.0183), but not EB alone (50%; *p* = 0.1032), displayed a surge when compared with controls (14%). In contrast, the percentage of C451A-ERα females reaching the surge threshold was low following both EB alone (0%) or EB + P (38%) such that no significant difference was found compared with the control condition (veh + veh, 18%; vs EB, *p* = 0.4762; vs EB + P, *p* = 0.3864). Contrasting with the observation obtained following repeated blood sampling, these results indicate that only EB + P induces an LH surge in WT-ERα females, but not in C451A-ERα mice. However, the absence of a significant increase in LH concentration in wild-type females treated with EB, the low percentage of females presenting an LH in the EB and EB + P conditions in wild-type, and the difference in basal LH level between genotypes, which is explained by dysregulated negative feedback (Faure et al., Submitted), make these observations difficult to interpret.

**Figure 4.**
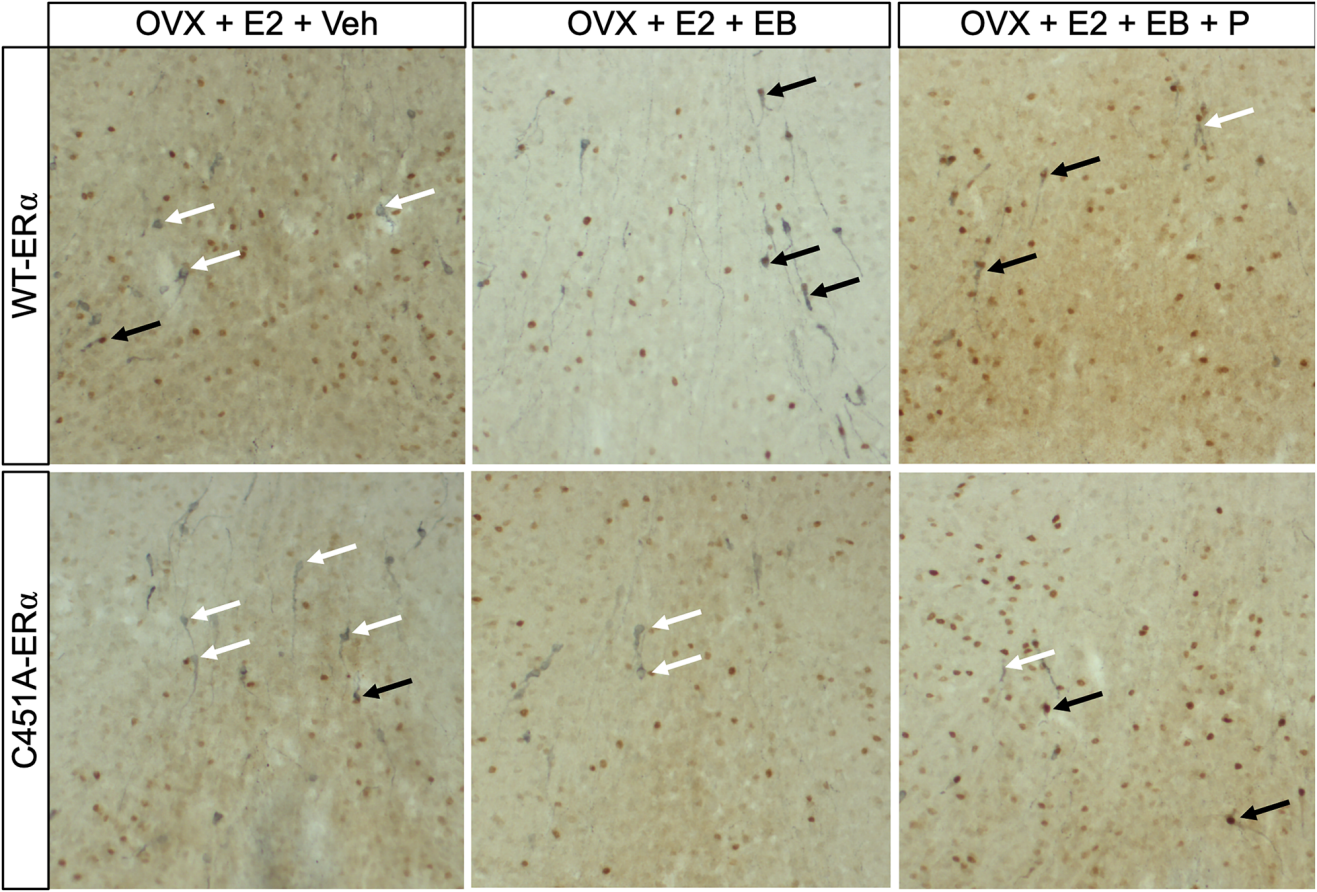
Representative photomicrographs of GnRH-IR neurons (in blue) and their coexpression of the neuronal activity marker Fos (in orange) as a function of the treatment and genotype. Black arrows point at double-labeled neurons, while white arrows point at single-labeled neurons.

The brains of these females were then immunostained for Kp (Fig. 3) and GnRH (Fig. 4) along with Fos to determine the effect of the mutation on the activation of the hypothalamic circuits underlying the LH surge (Clarkson et al., 2008; Gonzalez-Martinez et al., 2008). This neuronal response is considered a more reliable index of surge initiation than LH itself (Clarkson et al., 2023). The analysis of the total number of Kp neurons in the AVPv-PeN continuum revealed a reduced number of Kp neurons in C451A-ERα females compared with their WT-ERα littermates (*F*_(1,70) _= 38.61; *p* < 0.0001; $\eta_{p}^{2}$ = 0.355; Fig. 2*C*) and a trend toward an effect of treatment (*F*_(2,70) _= 3.124; *p* = 0.0502; $\eta_{p}^{2}$ = 0.082). There was however no interaction between the two factors (*F*_(2,70) _= 1.177; *p* = 0.3142; $\eta_{p}^{2}$ = 0.033). In contrast, GnRH neurons were slightly more abundant in the POA of C451A-ERα compared with WT-ERα mice (*F*_(1,69) _= 5.476; *p* = 0.0222; $\eta_{p}^{2}$ = 0.074; Fig. 2*E*), but there was no effect of treatment (*F*_(2,69) _= 0.3376; *p* = 0.7147; $\eta_{p}^{2}$ = 0.010) or interaction between the two factors (*F*_(2,69) _= 1.976; *p* = 0.1463; $\eta_{p}^{2}$ = 0.054).

The analysis of the percentage of Kp and GnRH neurons colabeled with Fos revealed a very different pattern of response between genotypes (Figs. 2–4). In WT-ERα, EB administered alone or along with P activated a higher percentage of Kp neurons. In contrast, only EB + P elicited such an increase in C451A-ERα females. A two-way ANOVA indeed identified a trend toward a genotype effect (*F*_(1,70) _= 3.735; *p* = 0.0573; $\eta_{p}^{2}$ = 0.051), as well as a treatment effect (*F*_(2,70) _= 56.88; *p* < 0.0001; $\eta_{p}^{2}$ = 0.619) and an interaction between the two factors (*F*_(2,70) _= 11.17; *p* < 0.0001; $\eta_{p}^{2}$ = 0.242; Figs. 2*D*, 3). This interaction is explained by the significant effect of EB and EB + P compared with veh + veh in WT-ERα females but only EB + P induced such an effect in C451A-ERα females as well as the higher proportion of colabeled Kp neurons induced by EB in WT-ERα females compared with C451A-ERα females (see Fig. 2*D* for details).

Similarly, the percentage of GnRH neurons colabeled with Fos increased after EB alone and EB + P in WT-ERα females, while only EB + P resulted in such an increase in C451A-ERα females, which resulted in a genotype effect (*F*_(1,69) _= 8.481; *p* = 0.0048; $\eta_{p}^{2}$ = 0.109), a treatment effect (*F*_(2,69) _= 34.52; *p* < 0.0001; $\eta_{p}^{2}$ = 0.500), and an interaction between the two factors (*F*_(2,69) _= 3.458; *p* = 0.0371; $\eta_{p}^{2}$ = 0.091; Figs. 2*F*, 4). Similar to Kp neurons, this interaction is explained by the different pattern of response of C451A-ERα females to EB than WT-ERα females. Together, these results indicate that, while EB alone and EB + P activate Kp and GnRH neurons in WT-ERα females, only the EB + P combination mimics these effects in C451A-ERα females.

The percentages of activated Kp and GnRH neurons correlate with circulating LH concentrations in WT-ERα females treated with EB + P (Kp, *R* = 0.6314, *p* = 0.0206; GnRH, *R* = 0.8388, *p* = 0.0003), while it is not the case for the circulating LH in C451A-ERα females (C451A-ERα, Kp, *R* = 0.1253, *p* = 0.6835; GnRH; *R* = 0.1117, *p* = 0.7163; data not shown). This difference could be explained by the fact that brains and bloods were collected too early to detect the surge in most individuals. This interpretation goes along with the relatively low percentage of animals displaying a surge, regardless of treatment and genotype, but even less so in C451A-ERα females.

### Does E_4_ block the LH surge induced by estradiol benzoate (EB)?

This lack of activation of Kp and GnRH neurons in C451A-ERα females treated with EB alone but not with EB + P suggested that mERα signaling is required for the activation of the neural circuitry underlying LH surge generation by EB but that P can bypass the effect of mERα. This latter effect could be interpreted as an indirect confirmation of the role of mERα for neuroprogesterone synthesis and its pivotal role for the activation of this circuit. As E_4_ was described as an antagonist of mERα (Gérard et al., 2022), we wondered whether E_4_ could block the LH surge induced by EB and whether this effect could be prevented by P.

**Figure 5.**
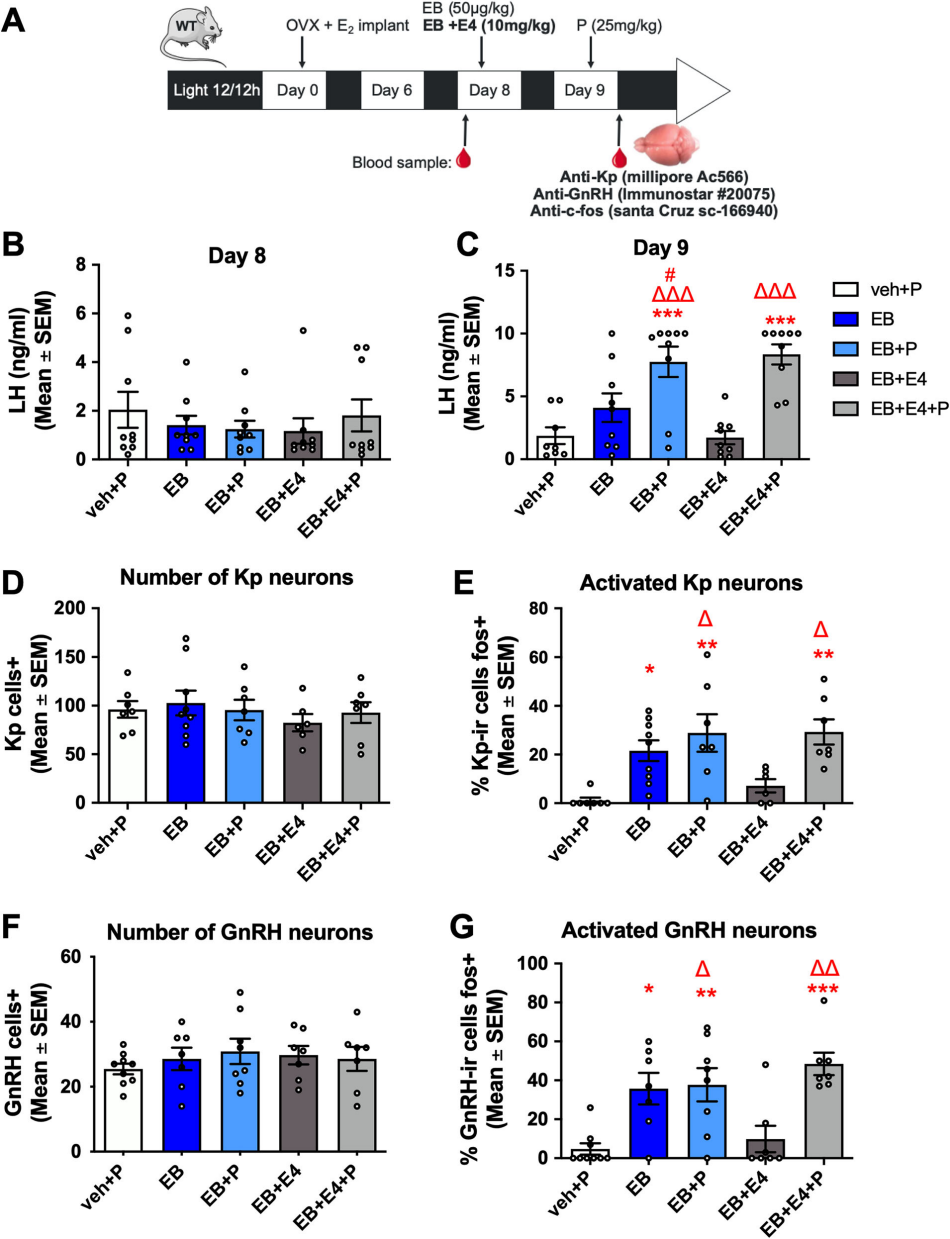
Effect of estetrol on the LH surge induced by estradiol and the neurocircuits underlying this response. ***A***, Protocol used to induce a positive feedback. WT mice were ovariectomized (OVX) on day 0, treated with subcutaneous estradiol (E_2_) implant from day 0 to day 9, and injected on day 8 with estradiol benzoate (EB) alone or combined with estetrol (E_4_, 200 µg, s.c.) or their vehicle (sesame oil) and on day 9 with progesterone (P) or its vehicle (sesame oil). Blood samples were collected prior to treatment on day 8 and within 1 h of lights off on day 9, when brains were also collected for immunohistological analyses. ***B***, LH levels did not differ between groups (*n* = 9) on day 8. ***C***, Females treated with EB alone (*n* = 9) or EB + E_4_ (*n* = 9) did not show a LH surge compared with veh + veh (*n* = 9) unless they were treated with P (EB + P, *n* = 9, and EB + E_4 _+ P, *n* = 9). ***D***, ***F***, The number of kisspeptin (Kp) neurons in RP3V (AVPv + PeN, ***D***) or GnRH neurons in POA (***F***) did not differ across treatments (Kp: veh + veh, *n* = 7, EB, *n* = 9, EB + P, *n* = 7, EB + E4, *n* = 6, EB + E4 + P, *n* = 7; GnRH: veh + veh, *n* = 9, EB, *n* = 8, EB + P, *n* = 9, EB + E4, *n* = 8, EB + E4 + P, *n* = 8). ***E***, ***G***, The percentages of Kp (***E***) and GnRH (***G***) neurons coexpressing Fos were higher in females treated with EB, EB + P, and EB + E_4 _+ P than females treated with veh + P and EB + E_4_ (same sample sizes as in ***D*** and ***F***). All data were analyzed by one-way ANOVA followed by Tukey’s post hoc test when significant: *, **, and *** *p* < 0.05, 0.01, and 0.001 versus veh + P; #, *p* < 0.05 versus EB; ^Δ^, ^ΔΔ^, and ^ΔΔΔ^, *p* < 0.05, 0.01, and 0.001 versus EB + E_4_.

This experiment followed a similar design as the second part of the previous experiment, except that in this experiment five treatments (veh + P, EB, EB + P, EB + E_4_, EB + E_4 _+ P) were compared in wild-type mice (Fig. 5*A*). As expected, LH levels assayed on samples collected before treatment (day 8) did not differ between groups (*F*_(4,40) _= 0.4620; *p* = 0.7631; $\eta_{p}^{2}$ = 0.044; Fig. 5*B*). In contrast, LH levels assayed within 1 h of lights off (28 h after treatment; day 9) were significantly elevated in females treated with EB + P and EB + E_4 _+ P compared with controls (veh + P), but not in females treated with EB + veh and EB + E_4_ (*F*_(4,39) _= 11.76; *p* < 0.001; $\eta_{p}^{2}$ = 0.547; Fig. 5*C*). Similarly, treatment with EB + P (77%; *p* = 0.0023) or EB + E4 + P (77%; *p* = 0.0023) led to a significantly higher percentage of females presenting a surge compared with controls (veh + P; 33%), while this was not the case for females treated with EB alone (0%; *p* = 0.2059) or combined with E4 (0%; *p* > 0.9999).

As previously, the brains of these females were immunostained for Kp or GnRH along with Fos to determine the effect of E_4_ on the activation of the hypothalamic circuits underlying the LH surge. The total number of Kp neurons in the AVPv-PeN continuum (Fig. 5*D*) and preoptic GnRH neurons (Fig. 5*F*) did not differ between treatments (Kp: *F*_(4,31) _= 0.4461, *p* = 0.7744, $\eta_{p}^{2}$ = 0.054; GnRH: *F*_(4,33) _= 0.4645, *p* = 0.7612, $\eta_{p}^{2}$ = 0.053) but the percentage of Kp and GnRH neurons expressing Fos differed between treatments (Kp, *F*_(4,31) _= 6.710, *p* = 0.0005, $\eta_{p}^{2}$ = 0.464; GnRH, *F*_(4,31) _= 8.489, *p* < 0.0001, $\eta_{p}^{2}$ = 0.507; Fig. 5*E–G*). These effects resulted from the significantly higher percentage of activated Kp and GnRH neurons compared with the control condition (veh + P) observed following the administration of all treatments with the exception of EB combined with E_4_.

Together, these results indicate that, in the absence of exogenous P, E_4_ prevents the activation of the neural circuit underlying the induction of an LH surge.

## Discussion

The present results indicate that, in the absence of P, a constitutive lack of mERα signaling as well as an acute treatment with E_4_ prevent the ability of E_2_ to activate Kp and GnRH neurons which are key neuronal populations for the LH surge generation. These pronounced effects (with $\eta_{p}^{2}$ comprised between 0.091 and 0.597 translating medium to large effect sizes) thus suggest a role for mERα in the activation of the neuronal circuit involved in the induction of the LH surge.

It should be noted however that the present data cannot extent this conclusion to the LH surge itself due to a lack of statistically significant LH surge in EB-treated WT females, despite numerous females showing higher LH than the average of the control group. cFos expression represents a transcriptional coupling to various types of stimuli, which reflects synaptic activation, accompanied or not by concurrent spike activity, mainly associated with an increased calcium influx and the activation of the MAPK pathway leading to the activation of the AP1 pathway and of late genes (Morgan and Curran, 1989; Luckman et al., 1994; Kovacs, 2008; Chung, 2015; Hudson, 2018). Increased cFos expression has long been used as a cell-specific marker of neuronal activity, notably in GnRH and Kp neurons in the context of LH surge induction (Hoffman et al., 1993; Clarkson et al., 2008, 2023; Dror et al., 2013). Transient cFos expression requires strong synaptic activation and is detected as a protein between 45 min and 3 h (peaking between 90 and 120 min; Kovacs, 2008). Such an extended time window of detection following stimulation leaves room for a mismatch between the measure of neuronal activation and the detection of a rise in LH. As previously shown, the amplitude of LH surge induced by EB alone is lower than this induced by the EB + P combination (Bronson and Vom Saal, 1979; Waring and Turgeon, 1992), which limits the detection of the surge. Moreover, the onset of LH surge is notoriously highly variable (Czieselsky et al., 2016). As blood samples were collected shortly after lights off, it is possible that LH surges of lower amplitude in this experimental group have been missed. Finally, C451A-ERα mice present elevated basal LH concentrations that could limit the detection of a surge in terminal blood. Therefore, given that the activation of Kp and GnRH neurons is considered as a reliable marker of the LH surge especially when only one blood collection is available (Clarkson et al., 2023) and the mechanisms underlying the positive and negative feedbacks operate independently from each other (Herbison, 2020; Goodman et al., 2022), the discussion of the present results will focus on the role of mERα in the activation of the neurocircuits underlying the induction of the LH surge rather than the surge itself.

### C451A-ERα females show a distinct phenotype of LH secretion

The idea that the positive feedback of estrogens depends on nuclear estrogen signaling is mainly based on the observation that the induction of a LH surge requires a prolonged exposure to high estrogen levels (Legan et al., 1975; Evans et al., 1997). Moreover, restoring ERE-independent ERα signaling had failed to restore the capacity to mount an LH surge in response to estrogens in ERαKO mice indicating that nonclassical signaling alone is not sufficient for positive feedback (Glidewell-Kenney et al., 2007). However, previous evidence supports the existence of a cooperation between nuclear- and membrane-initiated estrogen signaling (Vasudevan et al., 2001; Seredynski et al., 2013). Therefore, it is likely that membrane estrogen signaling requires nuclear estrogen signaling to properly function even if classical signaling constitutes the prime requisite for LH induction. To test this possibility, we used two complementary approaches. With a point mutation at the site of palmitoylation of ERα, C451A-ERα mice allow the study of the impact of a lack of membrane signaling of ERα while preserving its nuclear activity (Adlanmerini et al., 2014). Although this mutation does not seem to alter the sexual differentiation of females (Khbouz et al., 2019), the constitutive absence of mERα is an obvious limitation of such a model that may result in developmental defects and/or compensations. On the other hand, the antagonistic action of E_4_ on the membrane estrogen signaling of the classical estrogen receptors ERα (and possibly ERβ) provides a mean to circumvent developmental deficits or compensation. Although the preference of E_4_ for ERα over ERβ, GPER1, or STX-activated receptor as well as its properties in the brain (notably whether it activates or inhibits them) remain poorly documented, the comparison of effects obtained with both approaches provides confidence that membrane signaling plays a role in the activation of the circuit underlying the positive feedback of estrogens on LH secretion.

Gonadally intact C451A-ERα females exhibit elevated LH levels and fewer corpus lutea than wild-type females (Adlanmerini et al., 2014), suggesting a potential role of mERα signaling in both negative and positive feedbacks. The C451A mutation does not alter brain expression of ERα and Kp (Khbouz et al., 2019), suggesting the preserved transcriptional activity of the nuclear fraction of the receptor. The present study reveals however that C451A-ERα mice exhibit a distinct profile of LH secretion in response to estrogens compared with other ERαKO mouse models. Indeed, contrasting with ubiquitous ERαKO, neuron-specific ERαKO, and ARC specific ERαKO mice, which exhibit altered LH responses to ovariectomy and/or E_2_ (Wersinger et al., 1999; Cheong et al., 2014; Yeo and Herbison, 2014), C451A-ERα females respond to both ovariectomy (Fig. 1*B*) and provision of exogenous estrogens in the context of negative feedback (Faure et al., Submitted). However, C451A-ERα females are also unable to respond to increasing E_2_ levels by the activation of Kp and GnRH neurons (Fig. 2) which is typical following a surge induction protocol including P (Gonzalez-Martinez et al., 2008; Szymanski and Bakker, 2012). This is congruent with previous observations in other ERαKO or knockdown models which showed that the ubiquitous or neuron-/site-specific lack of ERα leads to impaired LH surge (Wintermantel et al., 2006; Cheong et al., 2015; Dubois et al., 2015; Porteous and Herbison, 2019; Wang et al., 2019). Interestingly, when treated with P, C451A-ERα females exhibit the typical activation of Kp and GnRH neurons (Fig. 2). Although one might wonder how the activation of the LH surge generation circuit is possible in mice showing such elevated circulating LH concentrations, this idea is compatible with the “two component model” of control of GnRH secretion which poses that the positive and negative feedbacks of gonadal steroids on LH regulation are regulated by two anatomically distinct and independent mechanisms (Herbison 2020). This idea is also supported by a recent study showing that a surge can be elicited over high LH levels (Chuon et al., 2022).

The inability of ovariectomized C451A-ERα females to show the characteristic activation of Kp and GnRH neurons by estrogens converges with the observation of reduced numbers of corpus lutea in gonadally intact C451A-ERα females (Adlanmerini et al., 2014) and suggests that this mutation leads to impaired ovulation and infertility. This was initially supported by the absence of pups in the nest of C451A-ERα females (Adlanmerini et al., 2014) and NOER females, another model generated following the same mutation of the palmitoylation site into an alanine (Pedram et al., 2014). However, a more careful investigation revealed that C451A-ERα females do get pregnant but lose their fetuses during the course of pregnancy and delivery, due to placental dysfunction and delayed labor induction, respectively (Rusidzé et al., 2022). Moreover, the ovulation rate of females mated overnight with a male did not differ between genotypes. This result is very surprising when compared with the present data as they indicate that ovary-intact C451A-ERα females are able to ovulate. Yet, it is important to consider that, when housed with females only, C451A-ERα females present irregular cycles with very rare estrous (they are essentially blocked in diestrus) suggesting very rare natural ovulations, matching the reduced number of corpora lutea. To assess ovulation rate, females were housed overnight with a male and mating was assessed by the presence of a vaginal plug, considered as an indication of estrous. Surprisingly, both WT and C451A-ERα females presented the same percentage of females with a plug on the next day (Rusidzé et al., 2022). A potential explanation is that exposure to male cues has overridden the blockade of the axis caused by the absence of mERα. Male cues are known to stimulate the activation of GnRH neurons (Taziaux and Bakker, 2015). In immature females, male cues induce estrus cycling and accelerate the cycle in adult group housed females (Whitten, 1956, 1958). However, this effect was reported to occur within 48 h, not overnight. This being said, in OVX mice chronically treated with a low dose of estrogens and hence presenting a high level of LH (similar to ovary-intact C451A-ERα females), the exposure to a male induces a rise in LH within 24 h (Bronson, 1976). Finally, overnight housing of acyclic aged female rats with a sexually active male led to a surge of LH secretion and ovulation whether they were allowed to copulate or not (Matt et al., 1987; Day et al., 1988). These effects are likely mediated by olfactory cues emitted by the males since exposure to male urine can restore ovulation in young females in persistent estrous (Johns et al. 1978). Interestingly, ovulation in aged females in persistent estrous cannot be mimicked by treatment with estrogens (Matt et al., 1987) and the reflexive LH surge elicited by male cues is associated with a rise of circulating progesterone concentration (Day et al., 1988). Together, these observations suggest the intriguing possibility that the absence of mERα from conception onwards may hamper spontaneous ovulation but somehow permit reflex ovulation in the presence of a mate. The mechanism underlying such an effect remains to be tested but would likely depend on the activation of GnRH and a rise of LH secretion following mating as in induced ovulators (Bakker and Baum, 2000).

### E_4_ acts as a mERα antagonist in the brain

E_4_ mimics the nuclear actions mediated by E_2_ on ERα in several tissues including the uterus (Abot et al., 2014), vagina (Benoit et al., 2017), the mammary gland (Gérard et al., 2015a), and cardiovascular system (Guivarc’h et al., 2018). Although antagonistic actions of E_4_ have been reported in several tissues including the brain (Pluchino et al., 2014; Gérard et al., 2015a,b; Pluchino et al., 2015), whether these effects are mediated by transcriptional or membrane ERα signaling is not known, with the exception of the membrane-mediated action identified in endothelial and breast cancer cells (Abot et al., 2014; Giretti et al., 2014) and in the brain (de Bournonville et al., 2023). The blockade of E_2_-induced activation of Kp and GnRH neurons by E_4_ in parallel with the absence of such response in mice lacking mERα signaling therefore provide converging evidence of the antagonist action of E_4_ on mERα in the brain and most probably within the preoptic area. However, the timing of these effects (estrogens being administered >24 h prior to sample collection) does not allow to determine whether they reflect direct membrane actions or membrane-initiated transcriptional effects. Further studies will be needed to identify the mechanism underlying these effects.

An alternative interpretation of these results is that E_4_ would act as an agonist of ER rather than an antagonist, thus exerting its effect through a negative feedback mechanism. However, it must be noted that E_4_ has a very short half-life in mice (2 h), contrasting with the situation in women (28 h; Gallez et al., 2023), making it unlikely that the injection received 34 h prior to sample collection could have resulted in a negative feedback effect that would have prevented the surge. Moreover, in a parallel study, a single dose of E_4_ induced a very moderate reduction of LH measured 3 h later, such that it does not reach statistical significance for several doses, including the one used in the present study, while chronic treatment with much lower doses resulted in a massive reduction in LH secretion (Faure et al., Submitted). Finally, it is important to note that E_4_ does not block the activation of Kp and GnRH neurons in the presence of the combination of EB and P, while P alone (Veh + P condition) does not activate these neuronal populations. As high estrogen levels are known to prevent LH induction by P (Bronson and Vom Saal 1979), this observation further argues in favor of an antagonist action of E_4_ and membrane ERα. In conclusion, although it cannot be ruled out that the absence of activation of Kp and GnRH neurons in mice treated with EB and E_4_ results from a negative feedback effect, this possibility seems unlikely. Future work targeting specific brain regions and neuronal populations is however necessary to confirm this hypothesis.

### Discrepancies between the two approaches

Although the two approaches employed in this study lead to similar conclusions, differences were observed. E_4_ altered EB-induced activation of Kp and GnRH neurons (Fig. 3) but had no impact on the number of Kp and GnRH neurons. In contrast, OVX and E_2_-treated C451A-ERα females exhibited elevated LH concentrations along with fewer Kp neurons and more GnRH neurons than their wild-type counterparts (Fig. 2). The presence of Kp in AVPv neurons of C451A-ERα mice confirms the preserved transcriptional activity of ERα, contrasting with the absent or greatly reduced Kp expression in the complete absence of ERα in Kp neurons (Smith et al., 2005; Gottsch et al., 2009; Dubois et al., 2015). However, the lower number of Kp neurons observed in the present experiment could be explained by a developmental effect of the constitutive mERα absence or by an effect of the mutation on Kp transcription. Although developmental defects cannot be ruled out, several observations points to the latter. First, the early programming of AVPv Kp neurons is affected by estrogen exposure in two critical periods. Perinatal exposure to estrogens leads to few detectable Kp neurons that are typical of males (Gonzalez-Martinez et al., 2008). In females, prepubertal exposure to estrogens is required to observe normal adult Kp neuronal numbers (Clarkson et al., 2009; Szymanski and Bakker, 2012; Brock and Bakker, 2013). Accordingly, C451A-ERα females exhibit expected numbers of Kp in the AVPv supporting an absence of programming defect in this cell population in females (Khbouz et al., 2019). Second, the number of Kp neurons in the AVPv of C451A-ERα females appears to be influenced by the dose of estrogens. Comparable numbers of Kp neurons were counted in the AVPv of wild-type and C451A-ERα females injected daily with EB (1 µg) for 2 weeks (Khbouz et al., 2019). Moreover, a parallel study found a difference between genotypes in females implanted with a Silastic capsule filled with 1 µg of E_2_ but not with a capsule containing 5 µg of E_2_ (Faure et al., Submitted). ERα may be less sensitive to estrogens in the mutant mice, thus requiring higher circulating concentrations of E_2_ to stimulate normal Kp expression as was recently shown to be the case in other tissues (Jiang et al., 2023). Finally, ERE-independent pathways are not sufficient to stimulate Kp expression in the AVPv of ERαKO mice (Gottsch et al., 2009). The lower number of Kp neurons in C451A-ERα mice thus seems attributable to a lower expression of Kp in the presence of low circulating estrogens. One report mentions, however, a stimulatory role for mERα in the expression of Kp in mHypo51A cells, an immortalized line derived from adult mouse hypothalamic neurons presumed to be AVPv Kp neurons (Mittelman-Smith et al., 2015).

### Role of progesterone signaling

Genetic or pharmacological blockade of mERα signaling prevented key neuronal populations for the induction of a LH surge by EB. In both cases, neuronal activation was restored by the administration of P 3–4 h before lights off. The potentiating effect of P on EB-induced surge has long been known (Bronson and Vom Saal, 1979). Its importance is underlined by studies focusing on progesterone receptors (PR), whose expression is stimulated by estrogens through an ERE-dependent genomic action (Moffatt et al., 1998). Knockout PR mice (PRKO) and mice lacking PR exclusively in Kp neurons (KissPRKO) are unable to mount an EB-induced surge (Chappell et al., 1999; Stephens et al., 2015; Gal et al., 2016). However, the reintroduction of PR expression specifically in Kp neurons of the AVPv of KissPRKO mice restores the LH surge, demonstrating the essential role of P action on this neuronal population for the induction of the LH surge (Mohr et al., 2021). Our results could thus suggest that the absence or blockade of mERα impedes PR expression. This hypothesis seems however unlikely given that C451A-ERα mice respond well to exogenous P in terms of Kp and GnRH activation. Moreover, E_4_ mimics the action of E_2_ on PR expression and E_2_ induces PR expression in the brains of C451A-ERα females, albeit to a lesser extent than in wild-type mice (Faure et al., Submitted). Membrane estrogen signaling could also interfere with another aspect of P signaling, such as its membrane-initiated or ligand-independent signaling (Tetel and Lange, 2009).

Alternatively, the present results support the notion that mERα modulates local P synthesis to contribute to LH surge induction (Micevych et al., 2015). Remarkably, all the studies supporting the necessity of PR to induce an LH surge, in particular within Kp neurons, did not provide exogenous P, suggesting that an endogenous source of P exists in OVX females which may be necessary to elicit the surge. This idea is supported by an absence of correlation between circadian fluctuations of brain and plasma P concentration in ovary-intact mice (Corpechot et al., 1997). Moreover, the work of Paul Micevych and his collaborators indicates that (1) a rise in neuroprogesterone produced by hypothalamic astrocytes is a prerequisite for the LH surge (Micevych et al., 2003; Micevych and Sinchak, 2008; Mohr et al., 2019; Chuon et al., 2022), (2) this rise depends on mERα activation (Micevych et al., 2007; Kuo et al., 2010; Mohr et al., 2022), and (3) neuroprogesterone’s action on LH is mediated by its action on Kp neurons (Mittelman-Smith et al., 2018). Therefore, it is possible that the lack of activation of the central pathway leading to LH surge in mice lacking mERα or following E_4_ treatment is explained by a blockade of hypothalamic P synthesis which is necessary for LH induction. In this model, mERα activation would thus stimulate neuroprogesterone synthesis by hypothalamic astrocytes and indirectly activate Kp neurons and in turn GnRH neurons.

### Conclusions

The present results contradict the idea that the central induction of a LH surge by rising concentrations of circulating estrogens is mediated by genomic effects only. Although it has long been known that the LH surge requires a prolonged exposure to high estrogen concentrations, it is also recognized that estrogens do not have to be present the whole time for the surge to occur (Legan et al., 1975; Evans et al., 1997). Moreover, membrane estrogen signaling through modulation of intracellular signaling cascades can potentiate the slower transcriptional actions of estrogens (Vasudevan et al., 2001; Kow and Pfaff, 2004). A role for membrane-initiated signaling in the induction of LH surge by estrogens is supported by the acute actions of E_2_ reported on the activity of GnRH neurons (Romano et al., 2008; Chu et al., 2009; Romanò and Herbison, 2012). It should also be pointed out that membrane-initiated signaling does not necessarily imply rapid actions, as indirect genomic signaling is also possible (Vasudevan and Pfaff, 2007). The present results cannot discriminate between these possibilities, nor can they determine the contribution of mERα located in the AVPv and ARC. Although it cannot be ruled out that the impaired positive feedback observed in mutant mice is an indirect result of the expected dysregulation of the negative feedback, this would not explain why Kp and GnRH neurons are still able to respond normally when provided with P along with EB. Moreover, the similarity of the responses of C451A-ERα mice to wild-type females treated with E_4_ supports a role for membrane-initiated estrogen signaling in the central induction of LH surge, probably through the activation of neuroprogesterone synthesis by hypothalamic astrocytes (Micevych et al., 2015). Further work will be necessary to identify where this contribution occurs.

## Synthesis

Reviewing Editor: Masha Prager-Khoutorsky, McGill University Decisions are customarily a result of the Reviewing Editor and the peer reviewers coming together and discussing their recommendations until a consensus is reached. When revisions are invited, a fact-based synthesis statement explaining their decision and outlining what is needed to prepare a revision will be listed below. The following reviewer(s) agreed to reveal their identity: NONE.

Synthesis of Reviews:

While the reviewers acknowledged that the manuscript has been improved, there are many remaining flaws. Reviewers expressed multiple concerns with regards to the experimental design and data interpretation. They also indicated that considering concerns with the methodology and experimental design, the findings are very incremental, and implication/conclusions are limited.

Some issues raised in the 1rst round of reviews remain unresolved or not thoroughly discussed. Accordingly, those issues as well as new ones raised in the 2nd round should be better discussed in the revised version. Furthermore, the limitations of conclusions and their implications should be better acknowledged in both the Abstract and Discussion.

---

Reviewer#1

The authors have addressed the reviews of their manuscript and have improved aspects of the manuscript with context to neuroprogesterone. The authors could set up as a hypothesis driven experiments given the more detailed introduction illustrating the actions of mERa and neuroprogesterone synthesis. However, the authors still make the initial paragraphs suggest that estradiol is directly acting on the Kp and GnRH neurons to "activate" them (as indicated by cfos expression).

Combined with the difficulty of the authors generating surge levels of LH, what does the "activation" that cfos reflect? Based on these data it does not reflect the production of an LH surge. The authors may want to consider using an alternate method than constant E2 dosing produced by the silastic implant. Do you know the circulating levels of E2 produced by the implant? Further, the constant prolonged exposure does not reflect the cyclic nature of the estrus cycle, which may alter the responsiveness subsequent E2.

The findings of the study are limited to mERa plays a role in the LH surge, and mERa mediates Kp and GnRH expression of cfos, but is not necessarily associated with generating an LH surge. These findings are somewhat new but very incremental.

mERa inhibition by E4 did not reduce EB induced LH levels, but did reduced cfos expression in GnRH and kp neurons. However, E4 did not affect subsequent progesterone actions indicating mERa actions are upstream of progesterone. Because of the inconsistent ability to produce robust LH surges in either the WT or mERaKO, and they still observe increased cfos expression in kp and GnRH neurons, these results brings into question what does it mean to be "activated" under these conditions that do not produce an LH surge?

Further, whether it is the same or different mER that induce cfos expression in Kp and GnRH neurons could have been tested. Where is it acting? These could have been part of the experimental design if due diligence would have been performed prior to designing and performing the experiments.

Work by John Lu and colleagues on rat estropause demonstrated that females in early persistent estrus could have their LH surges restored by exposure to a male, indicating that there is an alternate pathway to inducing the LH surge that appears to be reflexive to the male versus her usual spontaneous ovulation. These findings may be relevant to the mERaKO, which copulates, ovulates and becomes pregnant but does not maintain the pregnancy.

---

Reviewer#2

The authors have addressed several of the prior concerns and improved the manuscript. There are still some caveats with:

1) the KO mouse model (degree of KO still unknown, other unstudied phenotypic changes in the KO mice may contribute to the findings, the KO mice strain can ovulate and get pregnant so can generate LH surges),

2) the E4 drug possibly acting in their study as both antagonist and/or agonist (still unknown),

3) the brains and blood were collected at perhaps the wrong (non-peak LH surge) time for some groups in Figure 2 experiment, leading to possibly inaccurate conclusions about an "absent" activation of kisspeptin or GnRH neurons that may or may turn out not be the case if the brains were studied at a better (peak surge) time.

While those noted issues remain, some of the new rewording, additional discussion, and reorganization within the revised manuscript have improved it to some degree.

## Author Response

1. Using their model, the experimental results demonstrate that mERα are important for inducing the LH surge but could be rescued by progresterone treatment. These findings are not new. The authors have ignored the work of Micevych's lab and colleagues on the role of mERα in hypothalamic astrocytes in the induction of the neuroprogesterone synthesis. The authors do finally acknowledge the possibility of neuroprogesterone in the last paragraph of the discussion. However, what is more troubling is the lack of mentioning any of Micevych's findings that show mERα signaling and signaling pathways associated neuroprogesterone synthesis induction of the LH surge. The lack of these findings in the introduction indicates the lack of due diligence in performing a literature review prior to their experimentation and writing of the manuscript. The authors need to discuss and place their findings in context with these previous results. Their findings in the intact animals on E4 blocking ovulation parallel the effects of infusing aminoglutethamide (blocking neuroprogesterone synthesis) into the 3V of intact rats Mol Cel Endo; 2008 44-50. Thus, these findings also need to be put in context as well. The histological findings that mERα activation of GnRH and KP neurons are interesting but also need to put into the context of neuroprogesterone induction of the LH surge.

Although we agree that we should have presented in more details the extent of the work published by Paul Micevych and his collaborators and that we should have articulated better our findings with respect to theirs, we disagree with the conclusion that the present findings are not new. Paul Micevych and colleagues indeed showed that synthesis of progesterone in the hypothalamus is necessary for the LH surge, that membrane estrogen signaling induces neuroprogesterone synthesis by hypothalamic astrocytes and that pubertal changes in the content of neuroprogesterone coincide with LH surge capability. Yet, the role of membrane estrogen signaling on the activation of kisspeptin neurons and the subsequent activation of GnRH neurons had never been demonstrated in vivo. If we are not mistaken, the effects of membrane estrogen signaling on kisspeptin neurons was only conducted in vitro. Hence, our work is new and important as it actually provides in vivo support for the model developed by Paul Micevych. We have now modified the introduction to present our question in the context of this work and we discuss the present findings in the context of this model.

2. A major concern is that the results are not interpreted properly and the authors consistently over-reach in assigning a necessary role for mERa in the LH surge. The paper concludes that mERa is required for the LH surge but the findings show that LH surges actually occur in all genotypes and with/without E4. In fact, the results clearly demonstrate that females can generate an LH surge without mERa, as shown in EB+P females in both models. It seems the authors are forcing a necessary role of mERa in the LH surge when in fact their data show mERa is dispensable for surges to occur as long as the requisite hormonal milieu is present.

The manuscript focused on the central regulation of LH secretion, that is on the activation of kisspeptin and GnRH neurons, even if the initial targeted outcome was indeed LH itself. We thought our writing would make it clear enough that our conclusions relate to the central induction of the surge. This was for example clearly said in the following sentence that provided a potential explanation for the lack LH surge in estradiol treated females : "It should be noted however that due to a lack of LH surge in EB-treated females, likely explained by the fact that blood samples were collected too early after lights off, the present data cannot extent this conclusion to the LH surge itself"). Another explanation for LH levels not reaching statistical significance in EB treated females is the well-known high variability of LH surge onset and peak levels (Czielselsky et al., 2016). For this reason, we as others (see Clarkson et al.,https://doi.org/10.1101/2023.07.20.548652) considered cFos expression in GnRH neurons as a more reliable index of surge initiation than LH itself. This notion has been included in the text. Moreover, we have identified places in the manuscript where this notion could have been made clearer and have now clarified our focus on the activation of the circuit underlying the surge rather than on LH itself.

This being said, it is not true to say that the LH surge occurs in all genotypes and with and without E4. First, when considering LH, the data show no LH surge in EB treated mice but mice surged when P is provided, but not in all cases. For example, the LH profiles obtained in C451A-ERa mice following sacrifice are not suggestive of a surge (since they do not differ from the control condition in the same genotype, Fig 2B). Second, when considering the number of kisspeptin and GnRH neurons co-expressing cFos, a very different pattern of response emerges depending on the genotype (Fig 2D, 2F), even if not always fully statistically significant. While EB and EB+P both induced an increase in the number of double-labeled neurons in wild-type mice, only EB+P led to such a phenotype in C451A-ERa mice. Importantly, EB alone does not result at all in neuronal activation in this genotype (without mERa), contrasting with the wild-type situation. In addition, while the neuronal activation measured in wild-type mice correlates with LH levels in the EB+P condition, it is not the case in C451A-ERa mice (Fig 2D and F vs Fig 2B). The same applies for experiments testing the effect of E4. While EB alone resulted in increased cFos expression within Kp and GnRH neurons, this is not the case in the presence of E4, unless progesterone was administered (Fig 5E and 5G).

3. The author's conclusion that mERa females do not surge when given P is not entirely true since multiple mERa females showed surge-like LH and neuron activation, similar to controls. This was obscured in the group mean by the highly variable nature of the data in this genotype. In fact, several groups' data are very variable which limits interpretations.

We apologize but we do not see where this comment comes from. The data comparing terminal LH concentration and neuronal activation indeed suggest that C451A-ERa females do not show a statistically significant rise in LH surge whether treated with EB alone or EB+P (Fig 2B). This conclusion is corroborated by the analysis of the percentage of C451A-ERa females showing a rise in LH indicative of a surge (see new data added in the manuscript based on comment below (line 466)). Indeed, 62% of WT females treated with EB+P presented a surge (p = 0.0183) compared to controls (14%), while this was not the case for WT females treated with EB alone (50%, p = 0.1032). By contrast, the percentage of C451A-ERα females reaching the surge threshold was low following both EB alone (0%) or EB+P (38%) such that no significant difference was found compared to the control condition (veh+veh, 18%; vs EB, p = 0.4762; vs EB+P, p=0.3864). By contrast, the analysis of the activation of the neural pathway involved in surge generation indicates that these females are able to show the activation of the neuronal pathway involved in surge generation and this despite the high basal LH concentration they display (which does not differ from this of untreated females (fig 2D and 2F and is likely explained by the fact that this mouse model shows a dysregulated negative feedback (Fig 2B)). It is likely that these high basal LH levels are blurring the read out of the LH surge. Therefore, this observation along with the high variability in the initiation of the LH surge illustrated before indicates that the discussion should only focus on the effects observed in the brain and not so much on LH concentration.

4. This reviewer is familiar with the mERa knockout model in which there are unfortunately some phenotypic issues, including that the knockout is likely not complete (i.e., some tissue displays only a partial knockout, in about half the cells). This is disconcerting as it would greatly influence the present outcomes if not all cells, or only some cells, lack mERa. In addition, these transgenic mice exhibit a body weight phenotype with altered energy balance and metabolism. This is also a concern because fertility and kisspeptin systems are strongly affected by energy balance and body weight and different body weights between genotypes could affect hormone and brain outcomes.

Indeed, some authors argue that this ERa-C451A model (Adlanmerini et al. 2014) is not complete. We totally agree that we only reported ~60-70% reduction in membrane ERα expression in hepatocytes using sucrose gradient. We used this procedure since it was really difficult to visualize it at the plasma membrane by immunofluorescence due to its very low amount of membrane expression (Only 5%) and we can only visualize it in overexpressing conditions (Razandi et al., 2003). The result of the sucrose gradient did not demonstrate 100% loss of ERα expression in hepatocytes, but this procedure is quite difficult due to both the very low amount of ERα membrane expression and to the risk of contaminations between sucrose layers that could have contributed to alter our final results.

Two models with membrane loss-of-function (ERa-C451A) have been generated the same way, by mutating the palmitoylation site into an alanine to prevent membrane anchoring, and then membrane localization:, i.e; the ERa-C451A one published by Adlanmerini et al. (2014) used in this study and the NOER mice (Pedram A, Razandi M, Lewis M, Hammes S, Levin ER. Dev Cell. 2014). These two mouse models exhibit similar phenotypes in term of reproductive function (ovaries, fertility), except for the uterine response to E2. Indeed, in the C451A-ERα mouse model, the uterine response appears normal at the dose and time tested (0.01 mg/60 days at 24H), while in the NOER mice, the uterus is hypoplasic. The dose used to test the proliferative response in the NOER mice was very high (5mg/60 days) which could have largely impaired the proliferative response since the E2 response in the uterus follows a non-monotonic curve ((Fontaine et al., 2020). Moreover, the E2 uterine response of the same NOER mice used in the Gusfafsson's lab was not so dramatically affected and exhibited a 40 to 70 % decrease of uterine weight (Gustafsson et al., 2016).

For all these reasons, we believe that there is no rationale to believe that our model (ERa-C451A from Adlanmerini et al., 2014) is incomplete and differs from the NOER mice (Levin) since it is exactly the same mutation that has been introduced. We thus respectfully argue against the reviewer's comment suggesting that our mERa knockout is not complete. This ERa-C451A mouse model also lost acceleration of re-endothelialization and rapid vasodilatation that is strongly believed to be activated by membrane-initiated signaling and strongly cross-validated by other mouse models of membrane loss of function (Adlanmerini et al., 2020 Arteriosclr Thromb Vasc Biol 40:2143-2158) or pharmacological tools (Madak-Erdogan et al., 2016 Science signaling; 9:ra53).

5. The endocrine physiology of the mERa mice further imposes some limitations on the design and interpretations. There is a delay in the surge in the mERa knockouts, but this is not taken into account for the design or times of some of the tissue and blood collection. In those cases the two genotypes are not being compared at their own "peak" timing of the surge, as the authors acknowledge. This may present a group difference that is not really there since the groups are not at the same surge phase. In addition, the two genotypes have very different baseline LH which further restricts comparing the surge profile since mERa females start at an abnormally high LH level.

We agree that the impact of the mutation of ERa on both positive and negative feedback is a limitation, and we have not tried to hide it, but current knowledge also indicates that these two processes operate independently from each other (see recent work from the Herbison group). Obviously, if LH is the main endpoint, the endocrine physiology of this mouse model makes the data difficult to interpret, but as already pointed out this paper focuses on the activation of the neuronal circuit underlying this surge and this provides a clear readout.

As for the potential delay in the surge, this was not measured in a quantitative analysis, but was described qualitatively. All mentions of this delay, that we described as slight, was made using the conditional tense to express caution regarding this aspect. As the reviewer can see the temporal profile of LH levels shows high variability. Not all mice show a delay in LH peak. Demonstrating this would require additional work with many more mice. Therefore, we do not think this should be interpreted too strongly at this point. We have added verbiage in the text to clarify this aspect 6. There is a major concern with the use of E4 as a membrane receptor antagonist because this chemical is known to also have agonist actions on the nuclear receptor. Indeed, the literature (including its use as birth control) suggests as much, and the present results suggest that E4 is simply mimicking high E2 negative feedback and acting as an agonist instead of, or in addition to, acting as an antagonist. This dual agonist/antagonist property is therefore problematic, especially since these opposite actions also reportedly differ between cell types. On top of this critical concern, the E4 data do not conclusively identify a necessary role for mERa, contrary to the authors conclusions.

We realize that two types of manipulations have been presented in the present manuscript whose effects may be explained by different modes of action of E4 and may lead to confusion (original Fig 5 - Acute effect of E4 during positive feedback procedure vs original Fig 6 (Exp3) - Chronic effect of E4 on natural ovulation after mating in ovary intact mice). For this reason, we have decided to remove the data relative to the effect of multiple injections of E4 on ovulation (Exp3) and focus this manuscript on the complementary results obtained with the genetic and pharmacological approaches applied to the positive feedback paradigm. Although we cannot be sure that E4 behaves as an antagonist in this case, we recently provided evidence that E4 can act as an mERa antagonist in the brain (de Bournonville, Lemoine et al., 2023, J. Neuroendocrinol. 35(10):e13341). It is of course possible that this conclusion does not apply to the studied physiological response, as this property likely depends on the target cell type and its neurochemical make up. More work is thus needed to demonstrate one way or another. The alternative interpretation of the results proposed by the reviewer could thus be correct. However, it must be reminded that E4 has a very short half-life in mice (2h) which contrasts with the situation in women (28h) (Gallez et al., 2023 Int. J.Mol.Sci. 24:9718), so it seems quite unlikely that the injection received 34 hours prior to sacrifice could have resulted in a negative feedback effect that would have prevented the surge. This is all the more unlikely that the data of a parallel study (still unpublished) where we studied the acute effect of E4 on the negative feedback indicates that the effect of a single dose of E4 on LH measured 3 hours after treatment is quite moderate, such that it does not reach significance for several doses, including the one used in the present study. Of note, this contrasts with the effect of chronic E4 treatment (7 days of treatment) where much lower doses resulted in a massive reduction in LH secretion. Finally, it is important to note that in the presence of progesterone E4 does not block the activation of Kp and GnRH neurons, while P alone (Veh+P condition) does not activate these neuronal populations. As high estrogen levels are known to prevent LH induction by P (Bronson and Vom Saal 1979), this observation further argues in favor of an antagonist action of E4 on membrane ERa. In conclusion, although we cannot rule out the possibility that the absence of activation of Kp and GnRH neurons in mice treated with EB and E4 results from a negative feedback effect, we do not think it is the case. We have now added this aspect to the discussion.

7. In several cases the kisspeptin or GnRH neuron activation does not line up with the LH data, including in control mice in which these measures are known to correlate positively. This is problematic for making interpretations. Some of this may be due to the timing of the surge being different between genotypes but this possibility was not confirmed.

We are not sure we understand where this comment comes from as the pattern of neuronal activation follows the pattern of LH levels except for the C451A-ERa mouse for reasons that have been discussed above. Moreover, we showed that measures of neuronal activation do correlate with LH measures in wild-type but not C451A-Era mice. Although this lack of correlation in C451A-ERa mice may result from a delay in surge induction, this correlation is also likely to be blurred by the elevated basal LH concentrations in this genotype as discussed here above. So we believe that focusing on the circuit underlying the surge provides a much clearer picture.

8. A major concern is that a main control groups (EB only) did not produce LH surges as they should in any experiment, suggesting a technical or design problem. Because of this, the EB only analyses cannot be properly interpreted and the authors cannot compare EB only and EB+P groups since the former treatment did not work as intended.

Again, this is acknowledged in the manuscript and as discussed previously there are good reasons to explain this absence of effect and focus on the activation of the neuronal populations involved in surge generation as it allows to avoid the masking of some effects by elevated LH in this mouse model.

9. (Minor) What is the E4 treatment dose in Experiment 2? There is a wide range of E4 doses in Experiment 3 which is not well explained and the rationale for using those specific doses is not given.

We apologize for the confusion. This piece of information is located in the general method section about hormonal treatment. The dose was 200µg. It was chosen based on the 100-1000 fold weaker affinity and potency of E4 than E2 on ER (Abot et al., 2014).

10. (Minor) In addition to presenting LH levels, how many mice in each group surged (%) and was this different or similar between genotypes or groups? We have added this information in the text now (line 466).

11. (Minor) Are mERa mice fertile? If yes then mERa is not needed for the surge.

Although these mice were initially described as infertile (similar to the NOER mouse), a more careful investigation revealed that females do get pregnant but lose their offspring during the course of pregnancy and delivery, due to placental dysfunction and delayed labor induction respectively (Rusidzé, Faure et al., 2022 Development 149:dev200683). The quantification of ovulation rate in females mated overnight with a male did not reveal a difference in ovulation rate between genotypes. This result is very surprising when compared to the present data as they indicate indeed that these females are able to ovulate. But before rushing to a general conclusion, it is important to consider that these females present irregular cycles with very rare estrous (they are essentially blocked in diestrus), suggesting very rare natural ovulation. Interestingly, in the experiment setup we used, we did not experience more difficulties getting females to mate with a male (which we used as an indication that she is in estrous) as would be expected for females rarely entering estrous. The reason why is not clear, but one intriguing possibility would be that these females have become more sensitive to the male whose presence stimulated their entry in estrous. This is now discussed further in the paper.

## References

[B1] Abot A, et al. (2014) The uterine and vascular actions of estetrol delineate a distinctive profile of estrogen receptor alpha modulation, uncoupling nuclear and membrane activation. EMBO Mol Med 6:1328–1346. 10.15252/emmm.201404112 25214462 PMC4287935

[B2] Abraham IM, Han S-K, Todman MG, Korach KS, Herbison AE (2003) Estrogen receptor Beta mediates rapid estrogen actions on gonadotropin-releasing hormone neurons in vivo. J Neurosci 23:5771–5777. 10.1523/JNEUROSCI.23-13-05771.2003 12843281 PMC6741236

[B3] Acconcia F, Fiocchetti M, Busonero C, Fernandez VS, Montalesi E, Cipolletti M, Pallottini V, Marino M (2021) The extra-nuclear interactome of the estrogen receptors: implications for physiological functions. Mol Cell Endocrinol 538:111452. 10.1016/j.mce.2021.111452 34500041

[B4] Adlanmerini M, et al. (2014) Mutation of the palmitoylation site of estrogen receptor alpha in vivo reveals tissue-specific roles for membrane versus nuclear actions. Proc Natl Acad Sci U S A 111:E283–290. 10.1073/pnas.1322057111 24371309 PMC3896153

[B5] Alvisi RD, Diniz GB, Da-Silva JM, Bittencourt JC, Felicio LF (2016) Suckling-induced Fos activation and melanin-concentrating hormone immunoreactivity during late lactation. Life Sci 148:241–246. 10.1016/j.lfs.2016.02.038 26874026

[B6] Apter D, Zimmerman Y, Beekman L, Mawet M, Maillard C, Foidart JM, Coelingh Bennink HJ (2016) Bleeding pattern and cycle control with estetrol-containing combined oral contraceptives: results from a phase II, randomised, dose-finding study (FIESTA). Contraception 94:366–373. 10.1016/j.contraception.2016.04.015 27153745

[B7] Arnal JF, et al. (2017) Membrane and nuclear estrogen receptor alpha actions: from tissue specificity to medical implications. Physiol Rev 97:1045–1087. 10.1152/physrev.00024.2016 28539435

[B8] Bakker J, Baum MJ (2000) Neuroendocrine regulation of GnRH release in induced ovulators. Front Neuroendocrinol 21:220–262. 10.1006/frne.2000.0198 10882541

[B9] Balthazart J (2021) Membrane-initiated actions of sex steroids and reproductive behavior: a historical account. Mol Cell Endocrinol 538:111463. 10.1016/j.mce.2021.111463 34582978

[B10] Benoit T, et al. (2017) Estetrol, a fetal selective estrogen receptor modulator, acts on the vagina of mice through nuclear estrogen receptor α activation. Am J Pathol 187:2499–2507. 10.1016/j.ajpath.2017.07.013 28827141

[B11] Brock O, Bakker J (2013) The two kisspeptin neuronal populations are differentially organized and activated by estradiol in mice. Endocrinology 154:2739–2749. 10.1210/en.2013-1120 23744640

[B12] Bronson FH (1976) Serum FSH, LH, and prolactin in adult ovariectomized mice bearing silastic implants of estradiol: responses to social cues. Biol Reprod 15:147–152. 10.1095/biolreprod15.2.147 963145

[B13] Bronson FH (1981) The regulation of luteinizing hormone secretion by estrogen: relationships among negative feedback, surge potential, and male stimulation in juvenile, peripubertal, and adult female mice. Endocrinology 108:506–516. 10.1210/endo-108-2-506 7449740

[B14] Bronson FH, Vom Saal FS (1979) Control of the preovulatory release of luteinizing hormone by steroids in the mouse. Endocrinology 104:1247–1255. 10.1210/endo-104-5-1247 571329

[B15] Campbell RE, Herbison AE (2007) Definition of brainstem afferents to gonadotropin-releasing hormone neurons in the mouse using conditional viral tract tracing. Endocrinology 148:5884–5890. 10.1210/en.2007-0854 17823269 PMC6101187

[B16] Chappell PE, Schneider JS, Kim P, Xu M, Lydon JP, O’Malley BW, Levine JE (1999) Absence of gonadotropin surges and gonadotropin-releasing hormone self-priming in ovariectomized (OVX), estrogen (E2)-treated, progesterone receptor knockout (PRKO) mice. Endocrinology 140:3653–3658. 10.1210/endo.140.8.6895 10433223

[B17] Cheong RY, Czieselsky K, Porteous R, Herbison AE (2015) Expression of ESR1 in glutamatergic and GABAergic neurons is essential for normal puberty onset, estrogen feedback, and fertility in female mice. J Neurosci 35:14533–14543. 10.1523/JNEUROSCI.1776-15.2015 26511244 PMC6605465

[B18] Cheong RY, Porteous R, Chambon P, Abrahám I, Herbison AE (2014) Effects of neuron-specific estrogen receptor (ER) α and ERβ deletion on the acute estrogen negative feedback mechanism in adult female mice. Endocrinology 155:1418–1427. 10.1210/en.2013-1943 24476134

[B19] Christian CA, Glidewell-Kenney C, Jameson JL, Moenter SM (2008) Classical estrogen receptor alpha signaling mediates negative and positive feedback on gonadotropin-releasing hormone neuron firing. Endocrinology 149:5328–5334. 10.1210/en.2008-0520 18635656 PMC2584581

[B20] Chu Z, Andrade J, Shupnik MA, Moenter SM (2009) Differential regulation of gonadotropin-releasing hormone neuron activity and membrane properties by acutely applied estradiol: dependence on dose and estrogen receptor subtype. J Neurosci 29:5616–5627. 10.1523/JNEUROSCI.0352-09.2009 19403828 PMC2744362

[B21] Chung L (2015) A brief Introduction to the transduction of neural activity into Fos signal. Dev Reprod 19:61–67. 10.12717/DR.2015.19.2.061 27004262 PMC4801051

[B22] Chuon T, Feri M, Carlson C, Ondrejik S, Micevych PE, Sinchak K (2022) Progesterone receptor-Src kinase signaling pathway mediates neuroprogesterone induction of the luteinizing hormone surge in female rats. J Neuroendocrinol 34:e13071. 10.1111/jne.13071 34904297 PMC8923351

[B23] Clarkson J, Boon WC, Simpson ER, Herbison AE (2009) Postnatal development of an estradiol-kisspeptin positive feedback mechanism implicated in puberty onset. Endocrinology 150:3214–3220. 10.1210/en.2008-1733 19299459 PMC2703539

[B24] Clarkson J, d’Anglemont de Tassigny X, Moreno AS, Colledge WH, Herbison AE (2008) Kisspeptin-GPR54 signaling is essential for preovulatory gonadotropin-releasing hormone neuron activation and the luteinizing hormone surge. J Neurosci 28:8691–8697. 10.1523/JNEUROSCI.1775-08.2008 18753370 PMC6670827

[B25] Clarkson J, Herbison AE (2006) Postnatal development of kisspeptin neurons in mouse hypothalamus; sexual dimorphism and projections to gonadotropin-releasing hormone neurons. Endocrinology 147:5817–5825. 10.1210/en.2006-0787 16959837 PMC6098691

[B26] Clarkson J, Yip SH, Porteous R, Kauff A, Heather AK, Herbison AE (2023) CRISPR-Cas9 knockdown of ESR1 in preoptic GABA-kisspeptin neurons suppresses the preovulatory surge and estrous cycles in female mice. eLife 12:RP90959. 10.7554/eLife.90959.3 38126277 PMC10735218

[B27] Coelingh Bennink HJ, Skouby S, Bouchard P, Holinka CF (2008) Ovulation inhibition by estetrol in an in vivo model. Contraception 77:186–190. 10.1016/j.contraception.2007.11.014 18279689

[B28] Corpechot C, Collins BE, Carey MP, Tsouros A, Robel P, Fry JP (1997) Brain neurosteroids during the mouse oestrous cycle. Brain Res 766:276–280. 10.1016/S0006-8993(97)00749-X 9359616

[B29] Czieselsky K, Prescott M, Porteous R, Campos P, Clarkson J, Steyn FJ, Campbell RE, Herbison AE (2016) Pulse and surge profiles of luteinizing hormone secretion in the mouse. Endocrinology 157:4794–4802. 10.1210/en.2016-1351 27715255

[B30] Day JR, Morales TH, Lu JK (1988) Male stimulation of luteinizing hormone surge, progesterone secretion and ovulation in spontaneously persistent-estrous, aging rats. Biol Reprod 38:1019–1026. 10.1095/biolreprod38.5.1019 3408770

[B31] de Bournonville C, Lemoine P, Foidart JM, Arnal JF, Lenfant F, Cornil CA (2023) Role of membrane estrogen receptor alpha (ERalpha) in the rapid regulation of male sexual behavior. J Neuroendocrinol 35:e13341. 10.1111/jne.13341 37806316

[B32] Dror T, Franks J, Kauffman AS (2013) Analysis of multiple positive feedback paradigms demonstrates a complete absence of LH surges and GnRH activation in mice lacking kisspeptin signaling. Biol Reprod 88:146. 10.1095/biolreprod.113.108555 23595904 PMC4070868

[B33] Dubois SL, Acosta-Martínez M, DeJoseph MR, Wolfe A, Radovick S, Boehm U, Urban JH, Levine JE (2015) Positive, but not negative feedback actions of estradiol in adult female mice require estrogen receptor α in kisspeptin neurons. Endocrinology 156:1111–1120. 10.1210/en.2014-1851 25545386 PMC4330313

[B34] Duijkers IJ, Klipping C, Zimmerman Y, Appels N, Jost M, Maillard C, Mawet M, Foidart JM, Coelingh Bennink HJ (2015) Inhibition of ovulation by administration of estetrol in combination with drospirenone or levonorgestrel: results of a phase II dose-finding pilot study. Eur J Contracept Reprod Health Care 20:476–489. 10.3109/13625187.2015.1044082 26394847 PMC4673580

[B35] Evans NP, Dahl GE, Padmanabhan V, Thrun LA, Karsch FJ (1997) Estradiol requirements for induction and maintenance of the gonadotropin-releasing hormone surge: implications for neuroendocrine processing of the estradiol signal. Endocrinology 138:5408–5414. 10.1210/endo.138.12.5558 9389526

[B36] Franklin K, Paxinos G (2001) The mouse brain in stereotaxic coordinates, Ed 2. New York, NY: Academic Press.

[B37] Gal A, Lin PC, Cacioppo JA, Hannon PR, Mahoney MM, Wolfe A, Fernandez-Valdivia R, Lydon JP, Elias CF, Ko C (2016) Loss of fertility in the absence of progesterone receptor expression in kisspeptin neurons of female mice. PLoS One 11:e0159534. 10.1371/journal.pone.0159534 27441639 PMC4956300

[B38] Gallez A, et al. (2023) Comparison of estetrol exposure between women and mice to model preclinical experiments and anticipate human treatment. Int J Mol Sci 24:9718. 37298669 10.3390/ijms24119718 PMC10253893

[B39] Gérard C, et al. (2015a) Estetrol is a weak estrogen antagonizing estradiol-dependent mammary gland proliferation. J Endocrinol 224:85–95. 10.1530/JOE-14-0549 25359896

[B40] Gérard C, et al. (2022) Profile of estetrol, a promising native estrogen for oral contraception and the relief of climacteric symptoms of menopause. Expert Rev Clin Pharmacol 15:121–137. 10.1080/17512433.2022.2054413 35306927

[B41] Gérard C, Mestdagt M, Tskitishvili E, Communal L, Gompel A, Silva E, Arnal JF, Lenfant F, Noel A, Foidart JM, Péqueux C (2015b) Combined estrogenic and anti-estrogenic properties of estetrol on breast cancer may provide a safe therapeutic window for the treatment of menopausal symptoms. Oncotarget 6:17621–17636. 10.18632/oncotarget.4184 26056044 PMC4627333

[B42] Giretti MS, et al. (2014) Effects of estetrol on migration and invasion in T47-D breast cancer cells through the actin cytoskeleton. Front Endocrinol (Lausanne) 5:80. 10.3389/fendo.2014.00080 24904530 PMC4033260

[B43] Glidewell-Kenney C, Hurley LA, Pfaff L, Weiss J, Levine JE, Jameson JL (2007) Nonclassical estrogen receptor a signaling mediates negative feedback in the female mouse reproductive axis. Proc Natl Acad Sci U S A 104:8173–8177. 10.1073/pnas.0611514104 17470805 PMC1876590

[B44] Gonzalez-Martinez D, De Mees C, Douhard Q, Szpirer C, Bakker J (2008) Absence of gonadotropin-releasing hormone 1 and Kiss1 activation in alpha-fetoprotein knockout mice: prenatal estrogens defeminize the potential to show preovulatory luteinizing hormone surges. Endocrinology 149:2333–2340. 10.1210/en.2007-1422 18202134 PMC2329285

[B45] Goodman RL, Herbison AE, Lehman MN, Navarro VM (2022) Neuroendocrine control of gonadotropin-releasing hormone: pulsatile and surge modes of secretion. J Neuroendocrinol 34:e13094. 10.1111/jne.13094 35107859 PMC9948945

[B46] Gottsch ML, Navarro VM, Zhao Z, Glidewell-Kenney C, Weiss J, Jameson JL, Clifton DK, Levine JE, Steiner RA (2009) Regulation of Kiss1 and dynorphin gene expression in the murine brain by classical and nonclassical estrogen receptor pathways. J Neurosci 29:9390–9395. 10.1523/JNEUROSCI.0763-09.2009 19625529 PMC2819182

[B47] Guivarc’h E, et al. (2018) Predominant role of nuclear versus membrane estrogen receptor α in arterial protection: implications for estrogen receptor α modulation in cardiovascular prevention/safety. J Am Heart Assoc 7:e008950. 10.1161/JAHA.118.008950 29959137 PMC6064913

[B48] Hamilton KJ, Arao Y, Korach KS (2014) Estrogen hormone physiology: reproductive findings from estrogen receptor mutant mice. Reprod Biol 14:3–8. 10.1016/j.repbio.2013.12.002 24607249 PMC4777324

[B49] Herbison AE (1998) Multimodal influence of estrogen upon gonadotropin-releasing hormone neurons. Endocr Rev 19:302–330. 10.1210/edrv.19.3.0332 9626556

[B50] Herbison AE (2009) Rapid actions of oestrogen on gonadotropin-releasing hormone neurons; from fantasy to physiology? J Physiol 587:5025–5030. 10.1113/jphysiol.2009.179838 19687121 PMC2790245

[B51] Herbison AE (2020) A simple model of estrous cycle negative and positive feedback regulation of GnRH secretion. Front Neuroendocrinol 57:100837. 10.1016/j.yfrne.2020.100837 32240664

[B52] Herbison AE, Pape JR (2001) New evidence for estrogen receptors in gonadotropin-releasing hormone neurons. Front Neuroendocrinol 22:292–308. 10.1006/frne.2001.0219 11587554

[B53] Hoffman GE, Smith MS, Verbalis JG (1993) c-Fos and related immediate early gene products as markers of activity in neuroendocrine systems. Front Neuroendocrinol 14:173–213. 10.1006/frne.1993.1006 8349003

[B54] Holinka CF, Diczfalusy E, Coelingh Bennink HJ (2008) Estetrol: a unique steroid in human pregnancy. Climacteric 11[Suppl 1]:1. 10.1080/13697130802040077 18464015

[B55] Hudson AE (2018) Genetic reporters of neuronal activity: c-Fos and G-CaMP6. Methods Enzymol 603:197–220. 10.1016/bs.mie.2018.01.023 29673526 PMC6045948

[B56] Jiang Y, et al. (2023) Membrane estrogen receptor α signaling modulates the sensitivity to estradiol treatment in a dose-and tissue dependent manner. Sci Rep 13:9046. 37270592 10.1038/s41598-023-36146-9 PMC10239518

[B57] Johns MA, Feder HH, Komisaruk BR, Mayer AD (1978) Urine-induced reflex ovulation in anovulatory rats may be a vomeronasal effect. Nature 272:446–448. 10.1038/272446a0 565010

[B58] Kelly MJ, Rønnekleiv OK (2015) Minireview: neural signaling of estradiol in the hypothalamus. Mol Endocrinol 29:645–657. 10.1210/me.2014-1397 25751314 PMC4415204

[B59] Khbouz B, de Bournonville C, Court L, Taziaux M, Corona R, Arnal JF, Lenfant F, Cornil CA (2019) Role for the membrane estrogen receptor alpha in the sexual differentiation of the brain. Eur J Neurosci 52:2627–2645. 10.1111/ejn.14646 31833601

[B60] Klipping C, Duijkers I, Mawet M, Maillard C, Bastidas A, Jost M, Foidart JM (2021) Endocrine and metabolic effects of an oral contraceptive containing estetrol and drospirenone. Contraception 103:213–221. 10.1016/j.contraception.2021.01.001 33428907

[B61] Kovacs KJ (2008) Measurement of immediate-early gene activation- c-fos and beyond. J Neuroendocrinol 20:665–672. 10.1111/j.1365-2826.2008.01734.x 18601687

[B62] Kow L-M, Pfaff DW (2004) The membrane actions of estrogens can potentiate their lordosis behavior-facilitating genomic actions. Proc Natl Acad Sci U S A 101:12354–12357. 10.1073/pnas.0404889101 15302933 PMC514479

[B63] Kuo J, Hamid N, Bondar G, Prossnitz ER, Micevych P (2010) Membrane estrogen receptors stimulate intracellular calcium release and progesterone synthesis in hypothalamic astrocytes. J Neurosci 30:12950–12957. 10.1523/JNEUROSCI.1158-10.2010 20881113 PMC2957903

[B64] Legan SJ, Coon GA, Karsch FJ (1975) Role of estrogen as initiator of daily LH surges in the ovariectomized rat. Endocrinology 96:50–56. 10.1210/endo-96-1-50 1109905

[B65] Luckman SM, Dyball RE, Leng G (1994) Induction of c-fos expression in hypothalamic magnocellular neurons requires synaptic activation and not simply increased spike activity. J Neurosci 14:4825–4830. 10.1523/JNEUROSCI.14-08-04825.1994 8046453 PMC6577183

[B66] Matt DW, Coquelin A, Lu JK (1987) Neuroendocrine control of luteinizing hormone secretion and reproductive function in spontaneously persistent-estrous aging rats. Biol Reprod 37:1198–1206. 10.1095/biolreprod37.5.1198 3442697

[B67] McDevitt MA, Glidewell-Kenney C, Jimenez MA, Ahearn PC, Weiss J, Jameson JL, Levine JE (2008) New insights into the classical and non-classical actions of estrogen: evidence from estrogen receptor knock-out and knock-in mice. Mol Cell Endocrinol 290:24–30. 10.1016/j.mce.2008.04.003 18534740 PMC2562461

[B68] Memi F, Abe P, Cariboni A, MacKay F, Parnavelas JG, Stumm R (2013) CXC chemokine receptor 7 (CXCR7) affects the migration of GnRH neurons by regulating CXCL12 availability. J Neurosci 33:17527–17537. 10.1523/JNEUROSCI.0857-13.2013 24174685 PMC3812513

[B69] Micevych PE, Chaban V, Ogi J, Dewing P, Lu JK, Sinchak K (2007) Estradiol stimulates progesterone synthesis in hypothalamic astrocyte cultures. Endocrinology 148:782–789. 10.1210/en.2006-0774 17095591

[B70] Micevych P, Sinchak K (2008) Estradiol regulation of progesterone synthesis in the brain. Mol Cell Endocrinol 290:44–50. 18572304 10.1016/j.mce.2008.04.016 PMC2603025

[B71] Micevych P, Sinchak K, Mills RH, Tao L, LaPolt P, Lu JK (2003) The luteinizing hormone surge is preceded by an estrogen-induced increase of hypothalamic progesterone in ovariectomized and adrenalectomized rats. Neuroendocrinology 78:29–35. 12869797 10.1159/000071703

[B72] Micevych PE, Wong AM, Mittelman-Smith MA (2015) Estradiol membrane-initiated signaling and female reproduction. Compr Physiol 5:1211–1222. 10.1002/cphy.c140056 26140715 PMC4714864

[B73] Mittelman-Smith MA, Wong AM, Kathiresan AS, Micevych PE (2015) Classical and membrane-initiated estrogen signaling in an in vitro model of anterior hypothalamic kisspeptin neurons. Endocrinology 156:2162–2173. 10.1210/en.2014-1803 25730107 PMC4430613

[B74] Mittelman-Smith MA, Wong AM, Micevych PE (2018) Estrogen and progesterone integration in an in vitro model of RP3V Kisspeptin neurons. Neuroendocrinology 106:101–115. 10.1159/000471878 28384629 PMC5750133

[B75] Moenter SM, Chu Z (2012) Rapid nongenomic effects of oestradiol on gonadotrophin-releasing hormone neurones. J Neuroendocrinol 24:117–121. 10.1111/j.1365-2826.2011.02135.x 21496126 PMC3983560

[B76] Moffatt CA, Rissman EF, Shupnik MA, Blaustein JD (1998) Induction of progestin receptors by estradiol in the forebrain of estrogen receptor-alpha gene-disrupted mice. J Neurosci 18:9556–9563. 10.1523/JNEUROSCI.18-22-09556.1998 9801392 PMC6792867

[B77] Mohr MA, Esparza LA, Steffen P, Micevych P, Kauffman AS (2021) Progesterone receptors in AVPV kisspeptin neurons are sufficient for positive feedback induction of the LH surge. Endocrinology 162:1–8. 10.1210/endocr/bqab161 34379733 PMC8423423

[B78] Mohr MA, Keshishian T, Falcy BA, Laham BJ, Wong AM, Micevych P (2022) Puberty enables oestradiol-induced progesterone synthesis in female mouse hypothalamic astrocytes. J Neuroendocrinol 34:e13082. 10.1111/jne.13082 35000221 PMC9207152

[B79] Mohr MA, Wong AM, Tomm RJ, Soma KK, Micevych PE (2019) Pubertal development of estradiol-induced hypothalamic progesterone synthesis. Horm Behav 111:110–113. 10.1016/j.yhbeh.2018.12.007 30552874 PMC6527482

[B80] Morgan JI, Curran T (1989) Stimulus-transcription coupling in neurons: role of cellular immediate-early genes. Trends Neurosci 12:459–462. 10.1016/0166-2236(89)90096-9 2479148

[B81] Pedram A, Razandi M, Lewis M, Hammes S, Levin ER (2014) Membrane-localized estrogen receptor α is required for normal organ development and function. Dev Cell 29:482–490. 10.1016/j.devcel.2014.04.016 24871949 PMC4062189

[B82] Pluchino N, Drakopoulos P, Casarosa E, Freschi L, Petignat P, Yaron M, Genazzani AR (2015) Effect of estetrol on beta-endorphin level in female rats. Steroids 95:104–110. 10.1016/j.steroids.2015.01.003 25595451

[B83] Pluchino N, Santoro AN, Casarosa E, Giannini A, Genazzani A, Russo M, Russo N, Petignat P, Genazzani AR (2014) Effect of estetrol administration on brain and serum allopregnanolone in intact and ovariectomized rats. J Steroid Biochem Mol Biol 143:285–290. 10.1016/j.jsbmb.2014.04.011 24787659

[B84] Porteous R, Herbison AE (2019) Genetic deletion of Esr1 in the mouse preoptic area disrupts the LH surge and estrous cyclicity. Endocrinology 160:1821–1829. 10.1210/en.2019-00284 31145462

[B85] Romanò N, Herbison AE (2012) Activity-dependent modulation of gonadotrophin-releasing hormone neurone activity by acute oestradiol. J Neuroendocrinol 24:1296–1303. 10.1111/j.1365-2826.2012.02342.x 22612621

[B86] Romano N, Lee K, Abraham IM, Jasoni CL, Herbison AE (2008) Nonclassical estrogen modulation of presynaptic GABA terminals modulates calcium dynamics in gonadotropin-releasing hormone neurons. Endocrinology 149:5335–5344. 10.1210/en.2008-0424 18703628 PMC6116894

[B87] Rusidzé M, et al. (2022) Loss of function of the maternal membrane oestrogen receptor ERα alters expansion of trophoblast cells and impacts mouse fertility. Development 149:dev200683. 10.1242/dev.200683 36239412 PMC9720743

[B88] Seredynski AL, Balthazart J, Christophe VJ, Ball GF, Cornil CA (2013) Neuroestrogens rapidly regulate sexual motivation but not performance. J Neurosci 33:164–174. 10.1523/JNEUROSCI.2557-12.2013 23283331 PMC3710137

[B89] Smith JT, Cunningham MJ, Rissman EF, Clifton DK, Steiner RA (2005) Regulation of Kiss1 gene expression in the brain of the female mouse. Endocrinology 146:3686–3692. 10.1210/en.2005-0488 15919741

[B90] Stephens SB, Tolson KP, Rouse ML Jr, Poling MC, Hashimoto-Partyka MK, Mellon PL, Kauffman AS (2015) Absent progesterone signaling in kisspeptin neurons disrupts the LH surge and impairs fertility in female mice. Endocrinology 156:3091–3097. 10.1210/en.2015-1300 26076042 PMC4541622

[B91] Steyn FJ, Wan Y, Clarkson J, Veldhuis JD, Herbison AE, Chen C (2013) Development of a methodology for and assessment of pulsatile luteinizing hormone secretion in juvenile and adult male mice. Endocrinology 154:4939–4945. 10.1210/en.2013-1502 24092638 PMC5398599

[B92] Sun J, Chu Z, Moenter SM (2010) Diurnal in vivo and rapid in vitro effects of estradiol on voltage-gated calcium channels in gonadotropin-releasing hormone neurons. J Neurosci 30:3912–3923. 10.1523/JNEUROSCI.6256-09.2010 20237262 PMC2855130

[B93] Szymanski L, Bakker J (2012) Aromatase knockout mice show normal steroid-induced activation of gonadotrophin-releasing hormone neurones and luteinising hormone surges with a reduced population of kisspeptin neurones in the rostral hypothalamus. J Neuroendocrinol 24:1222–1233. 10.1111/j.1365-2826.2012.02334.x 22577852

[B94] Taziaux M, Bakker J (2015) Absence of female-typical pheromone-induced hypothalamic neural responses and kisspeptin neuronal activity in alpha-fetoprotein knockout female mice. Endocrinology 156:2595–2607. 10.1210/en.2015-1062 25860032

[B95] Terasawa E, Kenealy BP (2012) Neuroestrogen, rapid action of estradiol, and GnRH neurons. Front Neuroendocrinol 33:364–375. 10.1016/j.yfrne.2012.08.001 22940545 PMC3496051

[B96] Tetel MJ, Lange CA (2009) Molecular genomics of progestin actions. In: hormones, brain and behavior (Pfaff DW, Arnold AP, Etgen AM, Fahrbach SE, Rubin RT, eds), pp 1439–1465. San Diego, CA: Academic press.

[B97] Vasudevan N, Kow L-M, Pfaff DW (2001) Early membrane estrogenic effects required for full expression of slower genomic actions in a nerve cell line. Proc Natl Acad Sci U S A 98:12267–12271. 10.1073/pnas.221449798 11572951 PMC59803

[B98] Vasudevan N, Pfaff DW (2007) Membrane initiated actions of estrogens in neuroendocrinology: emerging principles. Endocr Rev 28:1–19. 10.1210/er.2005-0021 17018839

[B99] Wang L, Burger LL, Greenwald-Yarnell ML, Myers MG Jr, Moenter SM (2018) Glutamatergic transmission to hypothalamic kisspeptin neurons Is differentially regulated by estradiol through estrogen receptor α in adult female mice. J Neurosci 38:1061–1072. 10.1523/JNEUROSCI.2428-17.2017 29114074 PMC5792470

[B100] Wang L, DeFazio RA, Moenter SM (2016) Excitability and burst generation of AVPV kisspeptin neurons are regulated by the estrous cycle via multiple conductances modulated by estradiol action. eNeuro 3:ENEURO.0094-16.2016. 10.1523/ENEURO.0094-16.2016 27280155 PMC4895127

[B101] Wang L, Moenter SM (2020) Differential roles of hypothalamic AVPV and arcuate kisspeptin neurons in estradiol feedback regulation of female reproduction. Neuroendocrinology 110:172–184. 10.1159/000503006 31466075 PMC7047625

[B102] Wang L, Vanacker C, Burger LL, Barnes T, Shah YM, Myers MG, Moenter SM (2019) Genetic dissection of the different roles of hypothalamic kisspeptin neurons in regulating female reproduction. eLife 8:e43999. 10.7554/eLife.43999 30946012 PMC6491090

[B103] Waring DW, Turgeon JL (1992) A pathway for luteinizing hormone releasing-hormone self-potentiation: cross-talk with the progesterone receptor. Endocrinology 130:3275–3282. 10.1210/endo.130.6.1317780 1317780

[B104] Wersinger SR, Haisenleder DJ, Lubahn DB, Rissman EF (1999) Steroid feedback on gonadotropin release and pituitary gonadotropin subunit mRNA in mice lacking a functional estrogen receptor alpha. Endocrine 11:137–143. 10.1385/ENDO:11:2:137 10709760

[B105] Whitten WK (1956) Modification of the oestrous cycle of the mouse by external stimuli associated with the male. J Endocrinol 13:399–404. 10.1677/joe.0.0130399 13345955

[B106] Whitten WK (1958) Modification of the oestrous cycle of the mouse by external stimuli associated with the male; changes in the oestrous cycle determined by vaginal smears. J Endocrinol 17:307–313. 10.1677/joe.0.0170307 13587836

[B107] Wintermantel TM, et al. (2006) Definition of estrogen receptor pathway critical for estrogen positive feedback to gonadotropin-releasing hormone neurons and fertility. Neuron 52:271–280. 10.1016/j.neuron.2006.07.023 17046690 PMC6116893

[B108] Yeo SH, Herbison AE (2014) Estrogen-negative feedback and estrous cyclicity are critically dependent upon estrogen receptor-α expression in the arcuate nucleus of adult female mice. Endocrinology 155:2986–2995. 10.1210/en.2014-1128 24905671

[B109] Zhang C, Kelly MJ, Rønnekleiv OK (2010) 17 β-estradiol rapidly increases ATP-sensitive potassium channel activity in gonadotropin-releasing hormone neurons [corrected] via a protein kinase signaling pathway. Endocrinology 151:4477–4484. 10.1210/en.2010-0177 20660067 PMC2940490

